# Axonal endoplasmic reticulum tubules control local translation via P180/RRBP1-mediated ribosome interactions

**DOI:** 10.1016/j.devcel.2024.05.005

**Published:** 2024-08-19

**Authors:** Max Koppers, Nazmiye Özkan, Ha H. Nguyen, Daphne Jurriens, Janine McCaughey, Dan T.M. Nguyen, Chun Hei Li, Riccardo Stucchi, Maarten Altelaar, Harold D. MacGillavry, Lukas C. Kapitein, Casper C. Hoogenraad, Ginny G. Farías

**Affiliations:** 1 Cell Biology, Neurobiology and Biophysics, Department of Biology, Faculty of Science, Utrecht University, 3584 CH Utrecht, the Netherlands; 2 Biomolecular Mass Spectrometry and Proteomics, Bijvoet Center for Biomolecular Research and Utrecht Institute for Pharmaceutical Sciences, Utrecht University, 3584 CH Utrecht, the Netherlands; 3 Department of Neuroscience, Genentech, Inc., South San Francisco, CA 94080, USA

**Keywords:** axonal protein synthesis, mRNA localization, endoplasmic reticulum, ribosomes, P180/RRBP1, ER-based translation, axonal ER, local translation, neuron development, ER shape

## Abstract

Local mRNA translation in axons is critical for the spatiotemporal regulation of the axonal proteome. A wide variety of mRNAs are localized and translated in axons; however, how protein synthesis is regulated at specific subcellular sites in axons remains unclear. Here, we establish that the axonal endoplasmic reticulum (ER) supports axonal translation in developing rat hippocampal cultured neurons. Axonal ER tubule disruption impairs local translation and ribosome distribution. Using nanoscale resolution imaging, we find that ribosomes make frequent contacts with axonal ER tubules in a translation-dependent manner and are influenced by specific extrinsic cues. We identify P180/RRBP1 as an axonally distributed ribosome receptor that regulates local translation and binds to mRNAs enriched for axonal membrane proteins. Importantly, the impairment of axonal ER-ribosome interactions causes defects in axon morphology. Our results establish a role for the axonal ER in dynamically localizing mRNA translation, which is important for proper neuron development.

## Highlights

• Axonal ER tubules are required for proper mRNA translation in axons
• Nanoscale resolution imaging reveals ribosomes in contact with axonal ER tubules
• The axonal ribosome receptor P180 binds specific mRNAs and controls local translation
• Axonal ER-ribosome interactions are important for proper neuron development

## Graphical abstract

**Figure:**
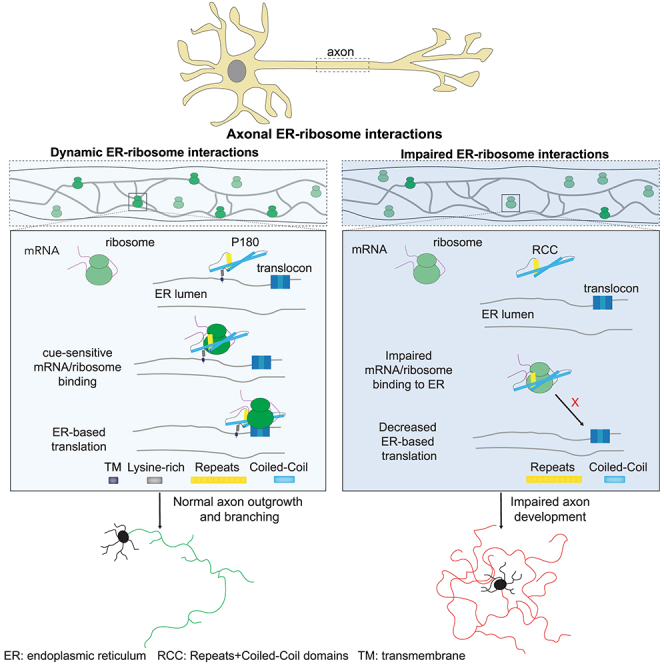

## Teaser

Koppers et al. use nanoscale resolution imaging to show that the axonal endoplasmic reticulum (ER) interacts with ribosomes to support ER-dependent local protein synthesis and identify P180/RRBP1 as an axonally distributed ER ribosome receptor that binds specific mRNAs. Disruption of axonal ER-ribosome interactions causes defects in axon morphology.

## Introduction

The complex and polarized nature of neurons requires precise regulation of the local proteome to maintain neuron development and function. mRNA localization and translation in dendrites and axons are essential for tight spatiotemporal control of the local proteome in response to local demands.^1^ Recent studies have shown that axons contain and translate a diverse set of mRNAs, often in response to extracellular signals, which is important for many neuronal processes, including axon guidance, branch/synapse formation, synaptic function, and survival.^2^^,^^3^^,^^4^ Despite recent progress on axonal mRNA transport and translation mechanisms,^5^ we still poorly understand where axonal protein synthesis takes place at a subcellular level and how this localization is achieved and regulated to fulfill local demands.

An unexplored player in local translation is the axonal endoplasmic reticulum (ER). Axons are devoid of rough ER or ER sheets and contain only ER tubules, which are presumed not to be involved in translation.^6^^,^^7^ However, studies in non-neuronal cells have shown that ribosomes, which bind ER sheets for translation of transmembrane, secretory, and a portion of cytosolic proteins,^8^ can also bind ER tubules.^9^^,^^10^ In addition, some ER proteins involved in translation, which normally reside on ER sheets, have been detected in axons.^11^^,^^12^ However, whether axonal ER tubules can bind ribosomes and have a functional role in local translation remains unknown.

Here, we establish a clear role for axonal ER tubules in the regulation of local mRNA translation in developing neurons. We demonstrate that the axonal ER interacts with ribosomes at the nanoscale resolution, and these contacts are sites for local translation in the axon. We reveal an extrinsic cue-specific regulation of these ER-ribosome contacts, suggesting an important role for the axonal ER in mediating stimuli-dependent translational regulation. We identify the integral ER protein P180 (also named RRBP1) as an axonally distributed ribosome/mRNA receptor, which facilitates ER-associated local translation. Mechanistically, P180 binds ribosomes in a mRNA-dependent manner through two binding sites, which together are sufficient to bind ribosomes and are required for efficient axonal ER-ribosome interactions. We find that P180 binds to specific mRNAs enriched for membrane and known axonally translated proteins. Importantly, we find that expression of a dominant-negative construct that impairs axonal ER-ribosome interactions leads to a reduction in the axonal translation of a specific mRNA and causes axon morphology defects. Together, our data indicate that the axonal ER, facilitated by P180, plays a critical role in regulating the subcellular localization of axonal mRNA translation.

## Results

### The axonal ER regulates local mRNA translation and ribosome levels in the axon

The ER appears continuous throughout the entire neuron, consisting of two distinct membrane organizations. ER sheets, decorated with polysomes, are distributed in the somatodendritic domain and excluded from the axonal domain, while ER tubules are present in both domains.^6^^,^^7^ Axonal ER tubules are distinct from somatodendritic ER tubules; just a few long and narrow tubules without much complexity have been observed along the axon.^13^

**Figure 1:**
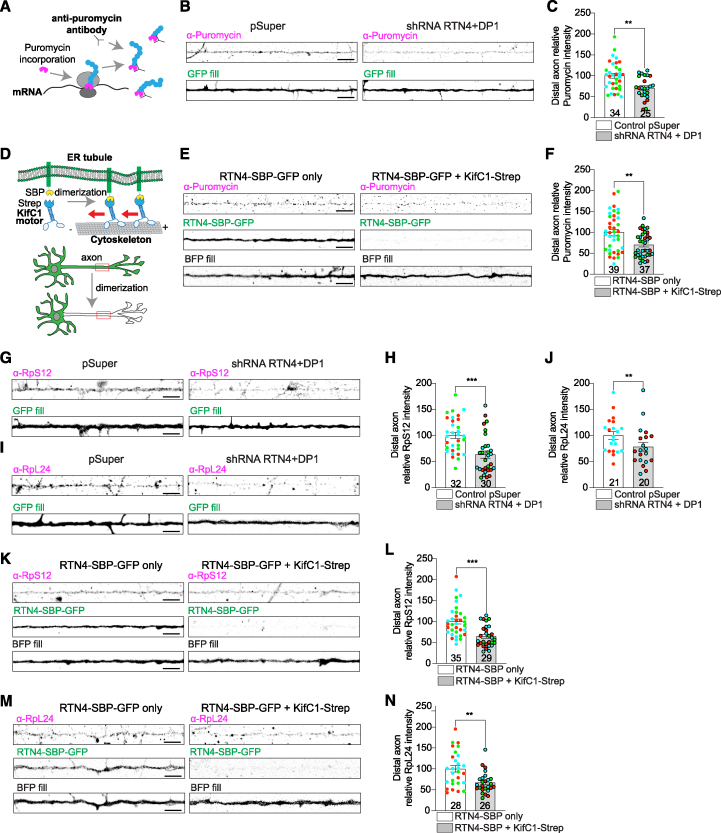
The axonal ER regulates local translation and ribosome levels (A–C) Schematic showing puromycin incorporation into newly synthesized proteins (A). Representative images of puromycilated peptides in distal axons of DIV7 neurons transfected with a fill and a pSuper plasmid (control) or pSuper plasmids containing shRNA targeting RTN4 and DP1 (B). Quantification of puromycin intensity (C). (D–F) Schematic showing streptavidin(Strep)-SBP heterodimerization system using SBP-RTN4 and Strep-KifC1 motor to relocate axonal ER into the soma (D). Representative images of puromycilated peptides in distal axons of DIV7 neurons expressing a fill and RTN4-SBP-GFP in absence or presence of Strep-KifC1 (E). Quantification of puromycin intensity (F). (G–J) Representative images of the distribution of endogenous ribosomal proteins RpS12 (G) and RpL24 (I) in the distal axon of neurons transfected as in (B). Quantification of RpS12 (H) and RpL24 (J) intensities from conditions as in (G) and (I), respectively. (K–N) Representative images of the distribution of ribosomal proteins RpS12 (K) and RpL24 (M) in distal axons of neurons transfected as in (E). Quantification of RpS12 (L) and RpL24 (N) intensities from conditions as in (K) and (M), respectively. Individual data points each represent a neuron, and each color represents an independent experiment. Data are presented as mean values ± SEM in (C), (F), (H), (J), (L), and (N). ^∗^*p* < 0.05, ^∗∗^*p* < 0.01, and ^∗∗∗^*p* < 0.001 comparing conditions to control with Mann-Whitney tests. Scale bars represent 5 μm. See also Figure S1 and Table S3.

To test the idea that the axonal ER is involved in local translation, we performed our experiments in primary cultures of rat hippocampal neurons at 7 days *in vitro* (DIV7), as most axonal translation research has been performed in growing/developing neurons with a well-defined axonal compartment (see STAR Methods and Koppers and Holt,^3^ van Beuningen et al.,^14^ Kaech and Banker,^15^ Martínez et al.,^16^ Kundel et al., and ^17^ Hengst et al.^18^). We first examined whether disruption of ER tubule formation affects axonal mRNA translation. Knockdown (KD) of the ER tubule-shaping proteins RTN4 and DP1 causes a drastic reduction of the axonal ER, which is retracted and inter-converted to ER sheets in the somatodendritic domain.^12^^,^^19^ Thus, we performed short hairpin RNA (shRNA)-mediated KD of RTN4 and DP1 (previously validated^12^^,^^19^) and labeled overall axonal translation using the puromycilation assay^20^ at DIV7. We incubated neurons for a short 10-min puromycin incubation, and newly synthesized proteins were labeled with an anti-puromycin antibody and quantified in a distal part of the axon, from regions ∼400–600 μm away from the soma (Figures 1A and S1A–S1C; see STAR Methods). This time frame is commonly used to detect local protein synthesis in dendrites or axons.^21^^,^^22^^,^^23^ Our axonal puromycin signal is unlikely to arise from diffusion from the soma in our primary neurons, as shown by only somatic or axonal puromycilation (Figures S1D–S1F). Compared with pSuper control neurons, RTN4/DP1 KD neurons showed a ±29% reduction in axonal puromycin labeling (Figures 1B and 1C). This decrease in axonal protein synthesis after KD is fully rescued by co-expressing RTN4 and DP1, confirming the specificity of our shRNAs (Figure S1G). This is a substantial reduction considering that translation on cytoplasmic, free ribosomes are not expected to be affected, and protein synthesis inhibitors cause a maximum of 70% reduction on axonal puromycilation (Figure S1B).^21^

Somatodendritic ER tubules are also disrupted by RTN4/DP1 KD.^19^ To study the local role of ER tubules in the axon, we used a previously validated heterodimerization system to remove ER tubules from the axon, measured by labeling different expressed and endogenous ER markers.^12^ In this system, a streptavidin module is coupled to the minus-end-directed motor KifC1 (Strep-KifC1) and a streptavidin binding protein (SBP) to GFP-tagged RTN4 (RTN4-SBP-GFP), which triggers their interaction and results in sustained removal of ER tubules from axons (Figures 1D and S1H).^12^^,^^19^ We found that puromycin intensity in the distal axon decreased by ±30% after axonal ER removal, similarly to RTN4/DP1 KD (Figures 1E and 1F). Axonal ER removal did not affect the amount of newly synthesized proteins in the soma (Figure S1I). These results show that axonal ER tubules contribute to local translation in the axon.

If the axonal ER is directly involved in translation, one would expect that disruption of axonal ER tubules would also affect ER-bound ribosomes and thus the number of ribosomes present in the axon. We therefore studied the distribution of ribosomes along the axon by labeling the endogenous proteins RpS12 (eS12) and RpL24 (eL24), which are mainly present in the small and large ribosomal subunits, respectively.^24^ We first disrupted ER tubules by RTN4/DP1 KD and quantified ribosomal proteins in the distal axon. Compared with pSuper control, RTN4/DP1 KD resulted in ±37% and ±22% fewer ribosomes in the axon based on RpS12 and RpL24 staining, respectively (Figures 1G–1J). We then specifically removed ER tubules from the axon, using the Strep-SBP heterodimerization system. Similar to RTN4/DP1 KD, the selective ER removal from the axon resulted in a significant decrease of both RpS12 and RpL24 in the axon (±35% and ±33% decrease, respectively) (Figures 1K–1N). Altogether, disruption of axonal ER tubules impairs translation and ribosome distribution along the axon, which suggests there may be a direct interaction between axonal ER tubules and ribosomes.

### Super-resolution imaging reveals that the axonal ER frequently contacts ribosomes in a translation-dependent manner

We next examined whether the role of axonal ER tubules in local translation is due to a direct contact of ER tubules with ribosomes. We set out to visualize the possible association between the axonal ER and ribosomes at nanoscale resolution, using three different super-resolution microscopy techniques.

**Figure 2:**
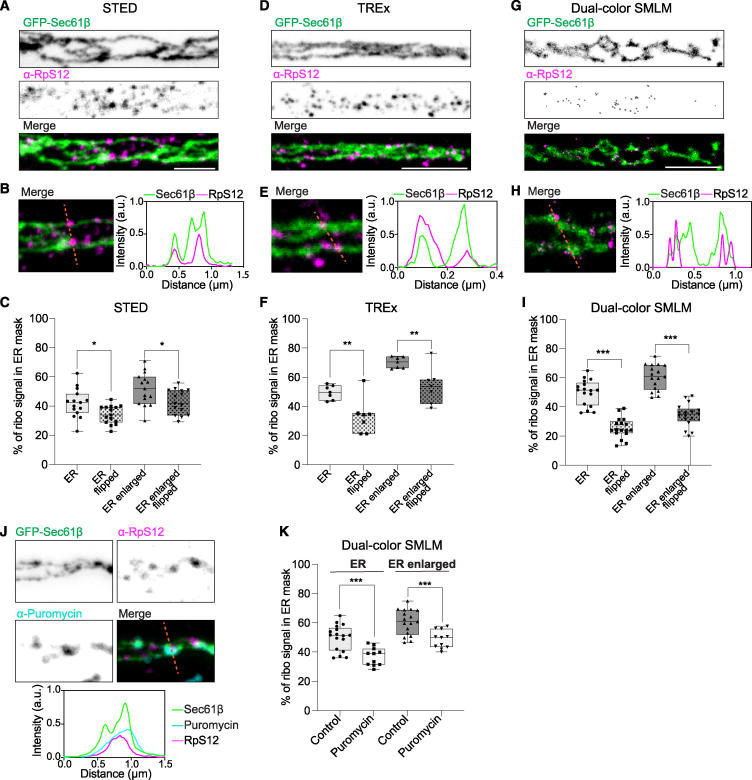
Nanoscale-resolved translation-dependent axonal ER-ribosome contacts (A, D, and G) Representative STED (A), TREx (D), and dual-color SMLM (G) images of the ER and ribosomes in axons from neurons expressing GFP-Sec61β and stained for RpS12. (B, E, and H) Magnifications and intensity profile lines from merged images for each microscopy method. (C, F, and I) Quantification of RpS12 intensity in ER mask, enlarged ER mask, and one-color flipped images, for each microscopy method. (J) Representative STED images and intensity profile line for an axon segment of a neuron transfected as in (A) and co-labeled for puromycilated peptides. (K) Quantification of RpS12 intensity in ER mask and enlarged ER mask with or without high puromycin treatment, using dual-color SMLM. For the control condition, the same neurons as in Figure 2I are used. Experiments were performed at the same time. Individual data points each represent a neuron in (C), (F), (I), and (K). Boxplots show 25/75-percentiles, the median, and whiskers represent min to max in (C), (F), (I), and (K). ns, not significant; ^∗^*p* < 0.05; ^∗∗^*p* < 0.01; and ^∗∗∗^*p* < 0.001 comparing conditions to control using unpaired t tests or ordinary one-way ANOVA tests. Scale bars represent 1 μm (A), (D), (G), and (J). See also Figure S2 and Table S3.

To this end, we expressed low levels of a tagged version of the general ER marker Sec61β. This marker is widely used to visualize the ER^25^ and is endogenously present in axons.^26^^,^^27^ We co-stained for the endogenous ribosomal protein RpS12. We first used stimulated emission depletion microscopy (STED), which allowed us to resolve axonal ER tubules, consistent with what has been observed with focused ion beam scanning electron microscopy (FIB-SEM) and cryo-electron tomography (cryo-ET),^6^^,^^28^ and generated a punctate signal for ribosomes (Figure 2A). Imaging of axonal segments and quantification revealed that ∼40%–50% of ribosomes are in close proximity to the axonal ER (Figures 2B and 2C). These contacts are not random since horizontally flipping one channel resulted in a significantly lower overlap (Figure 2C). To provide more robust evidence for this association, we utilized two other super-resolution techniques, ten-fold robust expansion microscopy (TREx)^29^ and dual-color single-molecule localization microscopy (SMLM), using probability-based fluorophore classification.^30^ Importantly, TREx and SMLM revealed a similarly high and specifically close association of ribosomes with the axonal ER (Figures 2D–2I and S2A). We investigated whether axonal ER-ribosome interactions also occur in mature neurons, by using STED. In mature neurons, axons are narrower than in DIV7 neurons, and the ER presented as a mostly single tubule, making it difficult to quantify enrichment of ribosomes at the ER. Nonetheless, STED imaging suggests that ribosomes may also be able to contact the ER in mature neurons (Figure S2B). Thus, three different super-resolution imaging techniques show consistent results at nanoscale resolution, indicating that a portion of axonal ribosomes are associated with the ER in axons.

Next, using STED, we visualized the axonal ER, ribosomes, and sites of translation, using the puromycilation assay. We observed various instances where ribosomes attached to the axonal ER were also positive for newly synthesized proteins (Figure 2J). This suggests that axonal ER-ribosome interactions are sites for local translation. However, two recent reports show that puromycilation may not accurately reflect the exact position of translation.^31^^,^^32^ Therefore, to further confirm that ribosomes bind the axonal ER in a translation-dependent manner and that the observed association is not random, we treated neurons with a high concentration of puromycin, which causes ribosome disassembly and release of small subunits from the ER.^25^^,^^33^ Visualization and quantification of dual-color SMLM revealed a significant decrease of RpS12 associated to the axonal ER after high puromycin treatment (Figure 2K). This is consistent with bona fide contacts between the axonal ER and ribosomes and, along with the reduction in axonal translation after axonal ER removal, indicates that the axonal ER is a subcellular site for local translation.

### Subcellular specificity and cue-selective regulation of axonal ER-ribosome interactions

**Figure 3:**
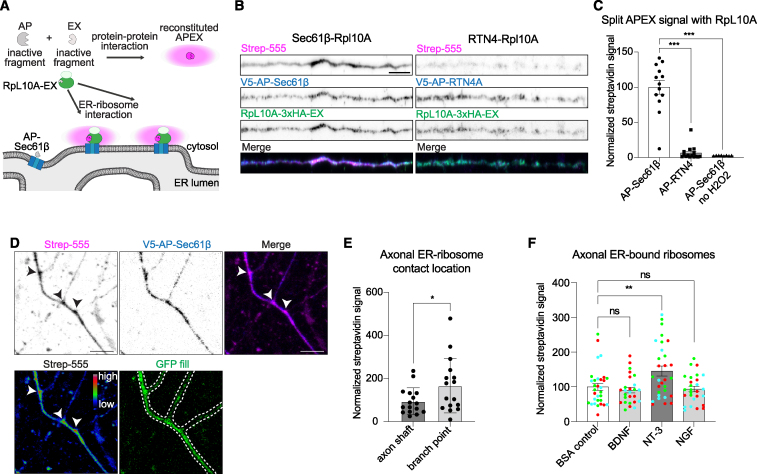
Axonal ER-ribosome interactions are enriched at axon branch points and regulated by extrinsic cues (A–C) Schematic representation of split APEX system used to detect ER-ribosome contacts. When the ER protein Sec61β fused to AP module and the ribosomal protein RpL10A fused to EX module interact with each other, APEX is reconstituted, and contact sites can be visualized as a biotinylation radius around the interactions (A). Representative images of split APEX assay in distal axons from neurons expressing RpL10A-3xHA-EX and V5-AP-Sec61β (left) or V5-AP-RTN4A as a negative control (right). Expression of constructs is visualized with V5 and HA antibodies, and biotinylation is detected with conjugated Strep-555 (B). Quantification of Strep signal in distal axons from neurons as in (B) and without H2O2 as a negative control for the biotinylation reaction (C). (D) Representative image of an axon with branches of a DIV7 neuron expressing V5-AP-Sec61β and RpL10A-3xHA-EX DIV7, showing enrichment of split APEX signal at branch points. White arrowheads indicate branch points. Split APEX signal (Strep555) is shown in magenta and a high-low intensity scale as indicated in lower panel. (E) Quantification of axonal ER-bound ribosomes, using split APEX assay as in (D), analyzed in the axon shaft and at axonal branch points. *n* = 16 per condition re-analyzed from six independent experiments. ^∗^*p* < 0.05 comparing conditions to each other using Mann-Whitney tests. Scale bars represent 5 μm. (F) Quantification of axonal ER-bound ribosomes, using split APEX assay with RpL10A-3xHA-EX and V5-AP-Sec61β, in neurons stimulated for 30 min with BSA (control), BDNF, NT-3, or NGF. Individual data points each represent a neuron in (C), (E), and (F), and each color represents an independent experiment in (F). Data are presented as mean values ± SEM in (C), (E), and (F). ns, not significant; ^∗^*p* < 0.05; ^∗∗^*p* < 0.01; and ^∗∗∗^*p* < 0.001 comparing conditions to control using unpaired t tests (C), Mann-Whitney test (E), or ordinary one-way ANOVA tests (F). Scale bars represent 5 μm (B) and (D). See also Figure S3 and Table S3.

Super-resolution imaging is not easily amenable to high-throughput quantifications in multiple conditions. For this reason, and to provide further evidence for a direct interaction, we utilized the split APEX system,^34^ previously adapted to visualize organelle-organelle contacts in neurons with known interacting proteins.^19^ Theoretically, ER-bound translating ribosomes bind the ER translocon, and we therefore attached an inactive AP-fragment to translocon subunit Sec61β and an EX-fragment to ribosomal protein RpL10A (uL1) (Figure 3A). We labeled RpL10A since it is well incorporated into ribosomes upon tagging.^35^^,^^36^^,^^37^ These two fragments only reconstitute and enable biotinylation of nearby proteins (10- to 20-nm labeling radius) when there is a molecular interaction between an ER protein and ribosomes. We first validated this system in neuronal soma where ER-bound ribosomes should be present. Quantification of somatic streptavidin labeling (which stains biotinylation sites) confirmed a specific interaction between Sec61β and RpL10A (Figures S3A and S3B). We then imaged axonal segments and quantified streptavidin labeling. Although the intensity of streptavidin labeling was expectedly lower than in the soma, we observed clear biotinylation with AP-Sec61β and RpL10A-EX in the axon. We did not observe such biotinylation when using RpL10A-EX in combination with AP-RTN4, which is an ER tubule protein not associated to ribosomes but abundant along the axonal ER, further confirming the specificity of this assay (Figure 3B). Quantification of axonal segments confirmed the specific contact between the ER and ribosomes in the axon (Figure 3C).

We noticed that our split APEX signal shows differences in intensities along the axon (Figure 3B), indicating there may be subcellular regions where axonal ER-ribosome interactions occur more often. Axon branch points are known hotspots for local translation,^38^^,^^39^ and we therefore quantified split APEX biotinylation at axon branch points, compared with the axon shaft (Figure 3D). Although we observed clear signal along the axon shaft, quantification revealed there are significantly more axonal ER-ribosome interactions occurring at axon branch points, with a 1.75-fold enrichment over interactions in the axon shaft (Figure 3E). This is consistent with observations in a recent cryo-ET study.^40^ Because of the morphology of the axon and the ER in mature neurons, STED imaging prohibited us from accurately assessing axonal ER-ribosome interactions in mature neurons. Therefore, we used split APEX, and this confirmed that the axonal ER contacts ribosomes in mature neurons (Figure S3C). In addition, like in developing neurons, split APEX signal seemed to be enriched at axon branch points and in putative synaptic boutons (Figures S3D and S3E).

Finally, we explored whether ribosome contacts with axonal ER tubules are regulated by neuronal stimuli. Extrinsic signals are well known to trigger and enhance axonal mRNA translation.^3^^,^^41^ We shortly stimulated neurons with brain-derived neurotrophic factor (BDNF), neurotropin-3 (NT-3), or nerve-growth factor (NGF) and quantified ER-ribosome contacts by using the split APEX assay. This revealed a cue-specific increase in axonal ER-ribosome interactions induced by NT-3 but not by BDNF or NGF (Figure 3F). Since it is well established that BDNF, NGF, and NT-3 can influence local translation, our data suggest that only NT-3 stimulation converges on ER-based translation during this time frame of stimulation.

Altogether, these results indicate that axonal ER-ribosome interactions occur along the axon shaft and are enriched at axon branch points, providing a possible way to achieve subcellular specificity of translation. In addition, we find that these interactions are regulated by specific neuronal stimuli. Therefore, we propose that the axonal ER serves as a platform for local protein synthesis in the axon that can provide subcellular specificity and can be dynamically regulated by extrinsic cues.

### P180 is an axonal ribosome receptor that facilitates axonal ER-ribosome interactions and local translation

**Figure 4:**
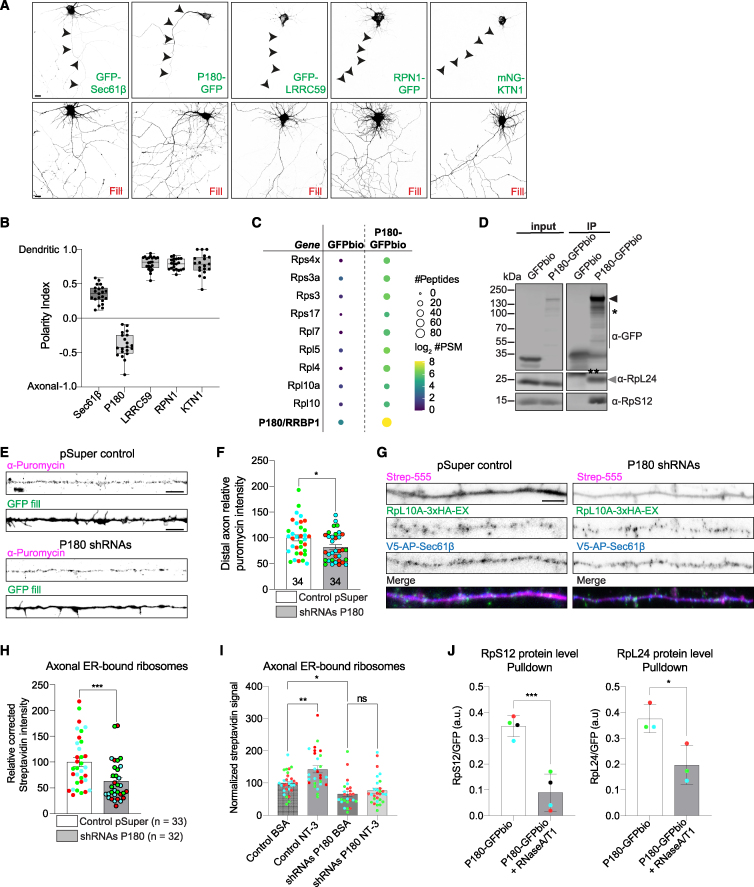
ER receptor P180 is enriched in axons and regulates local translation (A and B) Representative images of neurons expressing a fill and the ER-resident proteins GFP-Sec61β, P180-GFP, GFP-LRRC59, RPN1-GFP, or mNG-KTN1 (A), and respective quantification of polarity index (B). (C) Scaled representation of proteins identified with mass spectrometry after pull-down of GFPbio or P180-GFPbio from adult rat brain extracts. The size and color of each dot reflect the number of PSMs or peptides identified as indicated in the legend. (D) Western blot validation for ribosomal protein interactions with P180-GFPbio after streptavidin pull-down. Asterisk indicates that the presence of the coiled-coil domain in P180 causes protein instability, resulting in a banded pattern, as previously described.^44^ The double asterisk indicates an aspecific band at a different molecular weight than RpL24. (E and F) Representative images of puromycilated peptides in distal axons from neurons expressing a fill plus control pSuper plasmid or shRNAs targeting P180 (E). Quantification in (F). (G and H) Representative images of split APEX in distal axons for V5-AP-Sec61β and RpL10A-3xHA-EX, in the presence of control pSuper plasmid or shRNAs targeting P180 (G).Quantification in (H). (I) Quantification of axonal ER-bound ribosomes, using split APEX assay, in neurons expressing a pSuper control shRNA or shRNAs against P180 stimulated with BSA (control) or NT-3 for 30 min. (J) Western blot quantification of ribosomal proteins after GFPbio or P180-GFPbio pull-down with and without RNAseA/T1 treatment. Individual data points each represent a neuron (B), (F), (H), and (I) or an independent experiment (J). Each color represents an independent experiment. Data are presented as mean values ± SEM in (F), (H), (I) or ± SD in (J). Boxplots show 25/75-percentiles, the median, and whiskers represent min to max in (B) ^∗^*p* < 0.05, ^∗∗^*p* < 0.01, and ^∗∗∗^*p* < 0.001 comparing conditions to control using Mann-Whitney tests (F), (H), and (J) or one-way ANOVA test (I). Scale bars represent 10 μm (A) and 5 μm (E) and (G). See also Figure S4 and Tables S1 and S3.

We next sought to gain more insight into the proteins regulating axonal ER-ribosome interactions. Ribosomes can engage with the ER in signal recognition particle (SRP)-dependent and -independent manners.^42^^,^^43^ Besides the translocon, various ER-resident ribosome receptors have been proposed to play a role in ER-ribosome interactions in non-neuronal cells, and we therefore investigated the presence and/or enrichment of these proteins in the axonal compartment. We analyzed the subcellular distribution of four of these ER membrane proteins (P180/RRBP1, leucine-rich repeat-containing protein 59 [LRRC59], ribophorin 1 [RPN1]) (Figure S4A) and kinectin 1 (KTN1) as well as the translocon subunit Sec61β by quantifying the polarity index (PI; see STAR Methods). Consistent with our previous study^12^ and our super-resolution microscopy data, Sec61β had a mostly unpolarized distribution and was present in both dendrites and the axon (Figures 4A and 4B). P180 was present in the soma but enriched in the axon and nearly absent from dendrites, consistent with previous findings (Figures 4A and 4B).^12^ By contrast, LRRC59, RPN1, and KTN1 were strongly polarized toward the somatodendritic domain and were nearly absent from the axon (Figures 4A and 4B). In mature neurons, both Sec61β and P180 were also present in the axon (Figures S4B and S4C). The axonal distribution of Sec61β was confirmed at an endogenous level, using the CRISPR-Cas9 ORANGE system^45^ to endogenously label proteins (Figure S4D). Unfortunately, attempts to label endogenous P180 using this system were unsuccessful. The distribution of P180 in axonal ER tubules^12^ is different from its reported localization in ER sheets in non-neuronal cells^25^^,^^33^^,^^46^ and raises the interesting possibility that P180 plays a role in ribosome binding and translation in axons. We therefore decided to explore the role of P180 in these interactions and in axonal translation.

We first performed an unbiased screen of P180-interacting proteins by performing streptavidin pull-downs with GFP-biotin (GFPbio)-tagged P180 (Figure S4E) and subsequent mass spectrometry analysis using HEK293T lysates and adult rat brain extracts. In both cases, ribosomal proteins were among the top hits of interactors (Figures 4C and S4F; Table S1). Western blot analysis after streptavidin pull-downs further confirmed the interaction of ribosomal proteins with P180 (Figure 4D).

Although an interaction between ribosomes/mRNAs and P180, localized to ER sheets in non-neuronal cells, has been reported,^33^^,^^46^^,^^47^ a possible interaction of axonal P180 with ribosomes at ER tubules has not been shown. To investigate this, we used our split APEX assay with P180-AP and RpL10A-EX. This generated clear axonal biotinylation, suggesting P180 can interact with ribosomes in the axon (Figure S4H). We next wondered whether P180, enriched in axonal ER tubules and interacting with ribosomes, could play a role in the interaction between the axonal ER and ribosomes, thus regulating axonal protein synthesis. We therefore investigated the effect of previously validated shRNA against P180^19^ on axonal translation. P180 KD caused a reduction in axonal protein synthesis, as measured by puromycilation (Figures 4E and 4F). To confirm that this reduction was due to impaired contacts between the axonal ER and ribosomes, we used the split APEX assay with AP-Sec61β and RpL10A-EX. This revealed a significant reduction in ER-ribosome interactions in the axon after P180 KD (Figures 4G and 4H). Next, we investigated whether P180 is also required for the NT-3-induced increase in axonal ER-ribosome interactions. Split APEX quantification revealed that the significant NT-3-induced increase in interactions is blocked by P180 KD (Figure 4I).

Previous *in vitro* studies have suggested that P180 interacts with ribosomes through binding to mRNA.^33^^,^^48^^,^^49^ To determine if the interaction between P180 and ribosomal proteins is dependent on mRNA, we treated our pull-down samples with RNaseA/T1 to digest mRNA. This resulted in a significant decrease in ribosomal proteins bound to P180 (Figures 4J, S4I, and S4J), suggesting that the interaction of P180 with ribosomes is stabilized by its binding to mRNA. These data are consistent with a model in which P180 regulates the targeting of mRNAs and ribosomes to the axonal ER membrane, thereby regulating the translation of a subset of mRNAs.

### The cooperation of two domains in P180 and its association to the ER membrane are required for axonal ER-ribosome contacts

**Figure 5:**
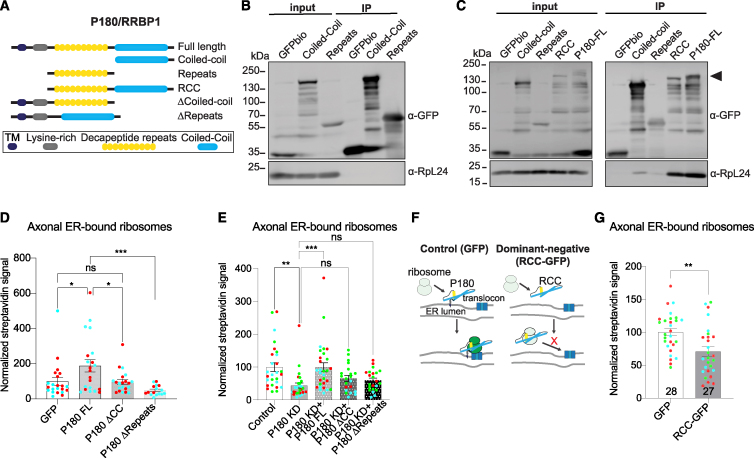
Axonal ER–ribosome interactions are regulated by specific P180 domains (A) Schematic representation of P180 constructs used. (B and C) Representative western blot analysis of GFP and endogenous RpL24 after GFPbio pull-downs with indicated constructs. The presence of the coiled-coil domain in P180 causes protein instability, resulting in a banded pattern, as previously described.^44^ (D and E) Quantification of axonal ER-bound ribosomes, using split APEX assay, in neurons expressing V5-AP-Sec61β and RpL10A-3xHA-EX, together with indicated P180 constructs and or shRNAs. (F and G) Schematic showing the dominant-negative effect of RCC-GFP in preventing ribosomes from associating with the ER and quantification of the effect of RCC-GFP on axonal ER-bound ribosomes using split APEX assay. Individual data points each represent a neuron, and each color represents an independent experiment. Data are presented as mean values ± SEM in (D), (E), and (G). ns, not significant; ^∗^*p* < 0.05; ^∗∗^*p* < 0.01; and ^∗∗∗^*p* < 0.001 comparing conditions to each other using ordinary one-way ANOVA tests (D) and (E), Mann-Whitney tests (G). See also Figure S5 and Table S3.

It remains unclear which domains of P180 are required for ribosome/mRNA binding.^33^^,^^44^^,^^46^ We attempted to resolve this by studying the binding site(s) and function(s) on axonal ER-ribosome contacts, using different P180 constructs (Figure 5A). We first performed GFPbio pull-downs with only the CC domain or only the repeats domain to see if one of these domains is the main binding site for ribosomes. However, pull-downs using only the CC or the repeats domain revealed very little to no interaction with ribosomes (Figures 5B and 5C). To determine whether the association of P180 to the ER membrane or the lysine-rich domain is required for P180-ribosome interactions, we expressed only the cytosolic domain containing both the repeats and CC domains (hereafter named RCC). Surprisingly, this construct, which is not localized to the ER, showed a strong interaction with ribosomes, similar to full-length P180 (Figure 5C). This shows that the repeats and CC domains together are necessary and sufficient to bind ribosomes.

To determine whether P180, associated to the ER membrane but lacking its CC or repeats domain, could interfere with the recruitment of ribosomes to the axonal ER, we performed the split APEX assay with AP-Sec61β and RpL10A-EX. We found that while full-length P180 promotes axonal ER-ribosome contacts, neither P180ΔCC nor P180ΔRepeats were able to induce ER-ribosome interactions (Figure 5D). Importantly, we evaluated the requirement of these two different domains for proper axonal ER-ribosome interactions. We found that the drastic reduction in axonal ER-ribosome contacts caused by P180 KD is fully rescued by full-length P180 but not by P180 lacking the CC or the repeats domain (Figure 5E).

Since the repeats and CC domains together are sufficient and required for P180-ribosome interactions, we wondered whether expression of both domains (RCC) could compete with the interaction of endogenous ER-associated P180 with ribosomes. Since this construct is not localized to the ER, it would thus specifically impair axonal ER-ribosome contacts. Indeed, we found that expression of RCC resulted in reduced axonal ER-ribosome contacts, using our split APEX assay with AP-Sec61β and RpL10A-EX (Figures 5F and 5G). Together, these results indicate that P180 is an axonal ribosome receptor with two essential binding sites required for proper ER-ribosome interactions.

### P180 interacts with specific mRNAs in neurons enriched in membrane protein-coding mRNAs

**Figure 6:**
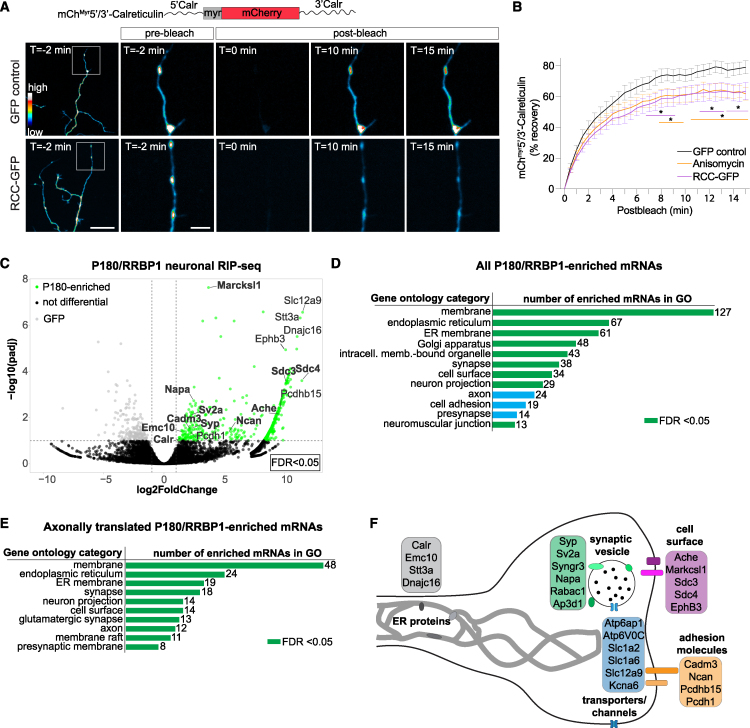
Axonal ER-ribosome interactions influence axonal calreticulin translation, and P180 interacts with specific mRNAs in neurons (A) Schematic diagram of the calreticulin FRAP reporter construct and representative pre- and post-bleach images of the calreticulin FRAP reporter in distal axons of DIV4 and -5 neurons. Scale bars represent 20 μm (left column) and 5 μm (right columns). (B) Quantification of FRAP assays in distal axons of mCh^Myr^5′/3′-Calreticulin (average % recovery) in GFP, RCC-GFP, and anisomycin-treated conditions. (C) Volcano plot showing differential gene expression analysis of mRNAs pulled down with P180, compared with GFP control. Gene names are indicated, and bolded names indicate that they have been previously detected in axonal translatome studies.^4^^,^^27^ (D) GO analysis of P180-enriched mRNAs identified by differential gene expression analysis. The number of genes in each category is plotted and noted after each bar. Green bars represent significantly enriched GO categories. (E) GO analysis of P180-enriched mRNAs that are also known to be axonally translated.^4^^,^^27^ The number of genes in each category is plotted and noted after each bar. Green bars represent significantly enriched GO categories. (F) Schematic representation of an axon/growth cone/pre-synapse highlighting known axonally translated mRNAs and their functional categories of mRNAs enriched after P180 pull-down. Line graph in (B) represents the mean ± SEM of recovery of 18 (GFP), 10 (RCC-GFP), or 12 (anisomycin) neurons per condition from two independent experiments. ∗*p* < 0.05 comparing conditions to each other using a 2-way ANOVA test. See also Tables S2 and S3.

Canonically, transmembrane, secretory, and luminal ER proteins are synthesized at the ER.^43^ Several transcriptome and functional studies have shown that the axon is capable of synthesizing proteins belonging to these functional categories.^4^^,^^27^ To gain first insights into which mRNAs could be translated at the axonal ER, we examined whether expression of the RCC dominant-negative domain affects the axonal translation of the luminal ER protein calreticulin, which was previously shown to be axonally translated.^50^^,^^51^ To test this, we fused 5′ and 3′ untranslated regions (UTRs) of calreticulin mRNA to the coding sequence of a diffusion-limited mCherry reporter cDNA (mCherry^myr^5′/3′-Calreticulin^50^; Figure 6A). Addition of this myristoylation epitope limits diffusion of the newly synthesized proteins from their site of translation.^52^ Fluorescence recovery after photobleaching (FRAP) provides a surrogate for localized translation of the reporter construct. We co-expressed mCherry^myr^5′/3′-Calreticulin with either GFP or RCC-GFP constructs and measured FRAP (Figure 6A). This revealed a significant reduction of axonal translation of mCherry^myr^5′/3′-Calreticulin upon RCC-GFP expression to levels comparable with protein synthesis inhibition (Figure 6B).

Next, to obtain more comprehensive insights into the possible mRNAs that could be translated at the axonal ER, we aimed to identify mRNAs specifically bound to P180. To achieve this, we performed RNA sequencing (RNA-seq) after immunoprecipitation (RIP-seq) of P180 in neurons. Differential gene expression analysis revealed 491 mRNAs specifically enriched after P180 pull-down (Figure 6C; Table S2). Consistent with the known role of the ER in the synthesis of membrane proteins, Gene Ontology (GO) enrichment analysis showed significant enrichment for the functional categories “membrane,” “ER membrane,” “neuron projection,” and “synapse” (Figure 6D). The enriched mRNAs included many mRNAs (142 out of 491; 28.9%) that were previously found to be translated in axons^4^^,^^27^ (bolded in Figure 6C). GO analysis of these 142 axonally translated P180-enriched mRNAs again showed significant enrichment for the functional categories membrane, ER membrane, neuron projection, and synapse (Figures 6E and 6F). Interestingly, these mRNAs included calreticulin and mRNAs coding for proteins important for neurite outgrowth/cell adhesion (marcksl1, sdc3, sdc4, and ncan), ion/amino acid transport (ache, atp6ap1, slc1a1, and kcna6), and synaptic function (syp, sv2a, and napa). Altogether, this shows that P180 binds a specific subset of mRNAs, enriched in membrane proteins, for which their local translation could serve an important role for neuron development and function.

### Impairment of axonal ER-ribosome interactions leads to defects in neuron morphology and growth dynamics

**Figure 7:**
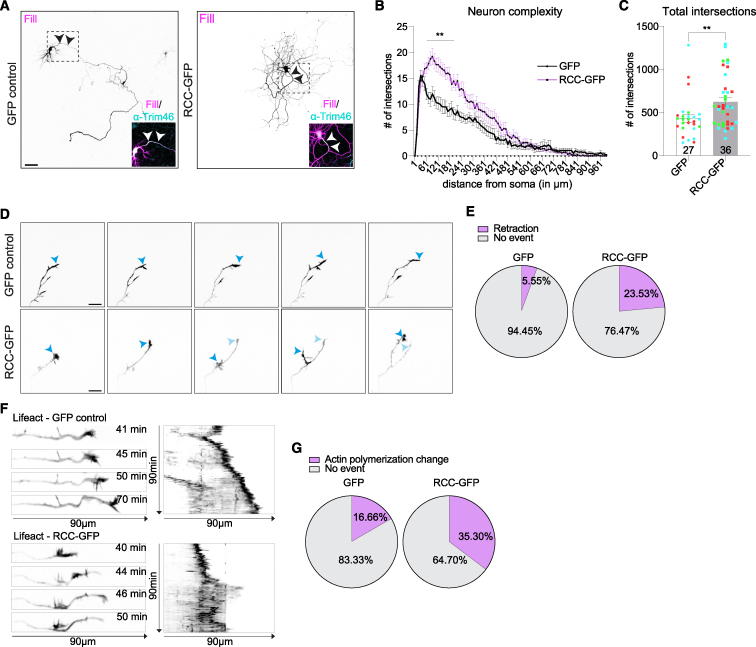
Disruption of axonal ER-ribosome interactions impairs axon growth and neuron morphology (A) Representative images of neurons at DIV7 transfected with mCherry fill and GFP or RCC-GFP and stained with a Trim46 antibody to identify the axon initial segment. (B and C) Sholl analysis of neuron complexity (B) and total intersections showing increased neuron complexity (C) in RCC-GFP-expressing neurons. (D and F) Still images (D) and kymographs (F) of DIV3 and -4 neurons transfected with GFP control or RCC-GFP together with LifeAct-mCherry to visualize growth cone dynamics. Blue arrowheads indicate the growth cone tip. Note the retraction and regrowth of a new growth cone and changes in actin flow in neurons expressing RCC-GFP. (E and G) Pie charts with the percentage of neurons per condition showing changes in retraction (E) or actin polymerization changes (G) in GFP control and RCC-GFP-expressing neurons. Individual data points each represent a neuron, and each color represents an independent experiment. Data are presented as mean values ± SEM in (C). Line graph in (B) represents the mean ± SEM of intersections of 27 (GFP) or 36 (RCC-GFP) neurons per condition. ns, not significant; ^∗^*p* < 0.05; ^∗∗^*p* < 0.01; and ^∗∗∗^*p* < 0.001 comparing conditions to each other using Mann-Whitney tests (C) or Mann-Whitney test with two-stage Benjamini, Krieger, and Yekutieli false discovery rate (FDR) procedure (B). Scale bars represent 50 μm in (A) and 5 μm in (D). See also Table S3.

Since we detected several P180-enriched mRNAs that can be locally translated and have a known function in neuron outgrowth or cell adhesion (Figures 6C–6E) and that neuron projection was a significantly enriched GO category (Figures 6E and 6F), we wondered whether the ER-ribosome interactions regulated by P180 influences neuronal development. To investigate this, we expressed GFP or the dominant-negative RCC-GFP and analyzed neuron morphology and complexity using Sholl analysis. We observed a drastic change in the morphology and directionality of axons (Figures 7A–7C), indicating a functional role of ER-ribosome contacts on proper neuron outgrowth and axonal morphology. Compared with control neurons, axons from RCC-expressing neurons seem to grow in a disorganized, non-directed manner, remaining closer to the soma (Figure 7A). Among the top enriched P180-bound mRNAs are various candidates that regulate axon guidance (Ephb3) or growth cone/filopodia dynamics (e.g., sdc3, sdc4, and marcksl1) (Figures 6C, 6E, and 6F). We therefore wondered if axon growth dynamics is affected by impairing axonal ER-ribosome interactions and examined this using live-cell imaging. To be able to analyze growth cone dynamics, we co-expressed Lifeact-mCherry^53^ together with GFP or RCC-GFP in neurons. In neurons expressing RCC-GFP, we observed disorganized growth with retraction of the growth cone and regrowth of a new growth cone into a different direction (Figure 7D). Consistent with this, we observed a higher frequency of changes in actin waves in RCC-expressing axons (Figure 7E). This change in actin dynamics included retrograde actin waves followed by new actin waves extending into a new branch (Figure 7F). This could underlie the disorganized axon morphology (Figure 7A). Altogether, this suggests that impairment of axonal ER-ribosome interactions leads to disorganized axon growth and defects in neuron morphology.

## Discussion

In this work, we find that axonal ER tubules play a key role in supporting local translation in developing neurons. The axonal ER binds ribosomes and is a site for local translation of at least a subset of mRNAs. The axonally enriched integral ER protein P180 acts as a ribosome/mRNA receptor that efficiently targets mRNA-bound ribosomes to the axonal ER for proper local translation. We propose a model in which P180 recognizes mRNA-containing ribosomes, enriched in membrane protein-coding mRNAs, by two binding sites present in its repeats and CC domains. Axonal ER-ribosome interactions provide a platform for efficient protein synthesis with the potential for subcellular specificity, and these contacts are dynamically regulated by specific extracellular stimuli. Finally, impairment of these interactions results in defective axon growth and neuron morphology.

Recent studies have shown that many mRNAs are present and being translated in the axon.^4^^,^^54^^,^^55^ Here, we show that axonal ER tubule disruption impairs overall local translation. The observed reduction in local translation (±30% decrease) suggests that only a fraction of all axonal translation occurs on the axonal ER. Using four different super-resolution techniques, we consistently find that ribosomes form contacts with the axonal ER in developing neurons. We also observe ribosomes attached to the axonal ER in mature neurons. Interestingly, we find that axonal ER-ribosome binding occurs along the axon shaft but is enriched at axon branch points. This provides a potential way for the ER to regulate the subcellular location of local translation. Consistent with our findings, a recent study has visualized clusters of ribosomes close to the ER at axon branch points by cryo-ET.^40^ In addition, another recent study provided some evidence for an association between the ER and ribosome in growth cones of motor neurons.^56^ The limited evidence of polysomes bound to axonal ER could be related to the possibility that ribosomes attached to the axonal ER consist of mainly monosomes, which are difficult to visualize by electron microscopy (EM) because of their small size. Monosomes were initially believed to be translationally inactive but were recently found to be actively involved in local translation, including translation of transmembrane, secretory, and cytosolic proteins.^55^ Our super-resolution imaging data unfortunately do not provide the resolution needed to dissect between polysomes and monosomes. It thus remains unclear whether they are bound to different regions of the axonal ER, for instance, polysomes at axon branch points and monosomes along the axon shaft. In addition, it has to be noted that it is possible that part of our split APEX signal is derived from interactions between the ER and only the large ribosomal subunit, although our super-resolution imaging data (Figure 2) support binding of the small subunit to the ER.

We identify P180 as an integral ER membrane protein that facilitates axonal ER-ribosome interactions and influences local translation. P180 was initially identified as a ribosome receptor located on ER sheets in non-neuronal cells^47^ but has also been suggested to target specific mRNAs to the ER, possibly via specific RNA-binding proteins (RBPs), thereby promoting ER-based translation.^48^^,^^49^ Indeed, we detected various RBPs in our P180 interactor screen (Figure S4G). Our findings of mRNA-dependent binding of ribosomes and P180-mediated recruitment of ribosomes to the ER are consistent with a model in which mRNA binding stabilizes ribosome interactions with the ER for local translation. Although we cannot formally rule out that the RNaseA/T1 treatment (Figure 4J) partially cleaves ribosomal RNA (rRNA) segments, thereby disrupting interactions that are rRNA mediated, our P180 RIP-seq data (Figures 6C–6F) further support this model. We find that the decapeptide repeats and coiled-coil domains together are required for efficient ribosome binding, and deletion of either of these domains impairs ribosome targeting to the axonal ER. Based on a predicted structure of P180, using Alphafold^57^ (Figure S5), it is possible that a region spanning both domains is essential for maintaining a structural conformation that allows efficient binding and targeting of mRNAs and ribosomes to the ER. Since P180 is also known to bind and stabilize microtubules (MTs),^12^^,^^58^ it is possible that P180 provides a molecular location at which MT-transported RNA granules can dock for subsequent mRNA translation at the axonal ER. In addition, P180 could limit translocon mobility by stabilizing MTs, which has been shown to increase ribosome binding to the ER.^59^

We find that specific extrinsic stimuli regulate axonal ER-bound ribosomes. Cue-specific changes in the axonal proteome are known to occur.^54^^,^^60^ Interestingly, a previous study identified a specific upregulation of several transmembrane protein mRNAs in the axon in response to NT-3, but not to BDNF or NGF, stimulation.^60^ Our data indicate that P180 is required for this NT-3-induced increase in axonal ER-bound ribosomes. Future studies are required to investigate the mechanisms underlying this cue-selective increase and how P180 is required for achieving this.

Importantly, we show that the axonal ER is a site for local translation. We find that axonal ER removal reduces axonal translation and axonal ribosomes. However, we cannot rule out that axonal ER removal also results in a decrease in axonal mRNA, and future research is needed to clarify this. We provide first insights into which mRNAs could be translated at the axonal ER. First, we showed that expression of the dominant-negative RCC domain affects the translation of calreticulin, a known axonally translated luminal ER protein.^50^ Next, we identified P180-bound mRNA. This revealed an enrichment of membrane protein-coding mRNAs bound by P180, suggesting the axonal ER is involved in axonal synthesis of membrane proteins. Consistent with this, a large portion of P180-enriched mRNAs have previously been shown to be axonally translated.^4^^,^^27^ The enriched mRNAs included calreticulin and various other interesting candidates, including axon guidance receptors (EphB3), cell adhesion molecules (e.g., ncan),^61^ and membrane-bound/secreted proteins important for neuron outgrowth (sdc3, sdc4, and marcksl1) (Figures 6E and 6F).^62^^,^^63^

Consistent with the functional importance of these axonally translated mRNAs, we find that expressing the RCC domain, which inhibits ribosome binding to the axonal ER, results in increased neuronal complexity and disrupted axon growth. RCC-expressing neurons seem to grow in a disorganized manner, and we observed changes in actin dynamics. In this respect, it is interesting to note that sdc3 and sdc4 were shown to be required for inhibition of neurite outgrowth,^62^ and marcksl1 influences actin dynamics and neuronal migration.^63^ It is therefore possible that disruption of the axonal translation of P180-enriched mRNAs may underlie the phenotypes seen after expressing RCC domains.

Future studies visualizing the translation of specific mRNAs are required to firmly establish the involvement of the axonal ER in transmembrane protein synthesis. Since P180 may not be the only protein regulating axonal ER-based translation, additional studies are needed to identify which exact mRNAs are being translated at the axonal ER. Altogether, this could reveal if the axonal ER is indeed involved mainly in the local synthesis of transmembrane/secretory proteins or if it can possibly also regulate the translation of mRNAs coding for cytosolic proteins, as these can also be translated on the ER membrane in non-neuronal cells.^8^^,^^64^

Previous studies have shown a role for lysosomes and mitochondria in axonal mRNA localization and translation through RNA granule hitchhiking.^21^^,^^65^^,^^66^^,^^67^ The ER forms contacts with many different organelles, including mitochondria and lysosomes, but also with RNA granules.^68^^,^^69^ We observe that ER tubule disruption reduces local translation, which is caused by impaired ribosome distribution. It is possible that a mislocalization of mitochondria or lysosomes in the axon, due to impaired contacts with the axonal ER, affects mRNA localization and translation. However, mitochondria or lysosome transport into the axon is not affected by axonal ER disruption.^19^ We still have a limited understanding of the possible role of organelle-organelle contacts in the distribution and dynamics of local translation in neurons. Our findings that the ER is involved in local mRNA translation opens the interesting possibility that ER-organelle contact sites are a location for mRNA exchange and translation between the ER and RNA granules or organelles on which RNA granules hitchhike.

Altogether, the results in this study indicate that the axonal ER plays a substantial role in regulating the local proteome. Although the functions of the axonal ER are poorly characterized,^70^ the axonal ER has been shown to be important for axon development and synaptic function.^12^^,^^71^ This work opens the possibility that local ER-based translation plays a supportive role in these processes. Our finding that impairment of axonal ER-ribosome interactions leads to defects in axon morphology is consistent with a role in neuron development. Interestingly, both ER dysfunction, partly through mutations in ER proteins,^72^ and dysregulation of local protein synthesis^73^ are reported in several neurological diseases. Our work provides a possible link between ER dysfunction and dysregulation of local translation in disease etiology.

### Limitations of the study

Our work is mostly limited to the visualization of overall translation, and our FRAP assay and RNA-seq data do not reveal the full contribution of the axonal ER to the axonal translatome. Although we provide evidence of ribosome interactions with the axonal ER in mature neurons, our study is mostly limited to developing neurons, and it will be of importance to study a possible role for axonal ER-based translation in mature neurons in the future. Finally, our findings are based on *in vitro*-cultured primary neurons, and future *in vivo* validation of our data will be important.

## STAR★Methods

### Key resources table

**Table:** 

REAGENT or RESOURCE	SOURCE	IDENTIFIER
**Antibodies**		

Mouse anti-Puromycin	Kerafast	EQ0001; RRID: AB_2620162
Rabbit anti-RpS12	Proteintech	16490-1-AP; RRID: AB_2146233
Rabbit anti-RpL24	Proteintech	17082-1-AP; RRID: AB_2181728
Rabbit anti-Trim46	Van Beuningen et al.^14^	N/A
Mouse anti-V5	Thermo Fisher Scientific	R960-25; RRID: AB_2556564
Rat anti-HA	Roche	11867423001; RRID: AB_390918
Mouse anti-GFP	Thermo Fisher Scientific	A-11120; RRID: AB_221568
Rabbit anti-GFP	Abcam	ab289; RRID: AB_303395
Streptavidin, Alexa-Fluor555 conjugate	Thermo Fisher Scientific	S21381; RRID: AB_2307336
Streptavidin, Alexa-Fluor568 conjugate	Thermo Fisher Scientific	S11226; RRID: AB_2315774
Goat-anti-Mouse Alexa Fluor 488	Thermo Fisher Scientific	A11029; RRID: AB_2534088
Goat-anti-Mouse Alexa Fluor 568	Thermo Fisher Scientific	A11031; RRID: AB_144696
Goat-anti-Mouse Alexa Fluor 405	Thermo Fisher Scientific	A31553; RRID: AB_221604
Goat-anti-Rabbit Alexa Fluor 647	Thermo Fisher Scientific	A21245; RRID: AB_2535813
Goat-anti-Rat Alexa Fluor 488	Thermo Fisher Scientific	A11006; RRID: AB_2534074
Goat-anti-Rabbit IRDye 800CW	Li-Cor	926-32211; RRID: AB_621848
Atto488 Fluotag-X4 GFP nanobody	NanoTag Biotechnologies	N0304-At488-L; RRID: AB_3075905
Goat-anti-Rabbit CF594	Sigma-Aldrich	SAB4600110
Goat-anti-Mouse STAR635P	Abberior	ST635P-1001; RRID: AB_2893232
Goat-anti-Mouse CF680	Biotium	20065; RRID: AB_10557108
Goat-anti-Rabbit Atto 647N	Sigma-Aldrich	40839; RRID: AB_1137669

**Chemicals, peptides, and recombinant proteins**		

Puromycin dihydrochloride	Sigma-Aldrich	P8833
Anisomycin	Sigma-Aldrich	9789
recombinant human Neurotrophin-3	Alomone Labs	N-260
recombinant human BDNF protein	Alomone Labs	B-250
recombinant rat beta-NGF protein	R&D Systems	556-NG
NeutrAvidin	Thermo Fisher Scientific	31000
Hemin (Heme)	Sigma-Aldrich	51280
Biotin-phenol	Iris Biotech	LS.3500
Hydrogen Peroxide	Sigma-Aldrich	H1009
Lipofectamine2000	Thermo Fisher Scientific	11668019
Polyethylenimine (PEI MAX)	Polysciences	24765
Fluoromount-G Mounting Medium	Thermo Fisher Scientific	00-4958-02
Dynabeads M-280 Streptavidin	Thermo Fisher Scientific	11205D
Pierce Streptavidin Magnetic beads	Thermo Fisher Scientific	88816
RNase A	Thermo Fisher Scientific	EN0531
RNase T1	Thermo Fisher Scientific	EN0541
SUPERase-in RNase Inhibitor	Thermo Fisher Scientific	AM2694
Trolox	Sigma-Aldrich	238831
Sodium L-Ascorbate	Sigma-Aldrich	A4034
Sodium Azide	Sigma-Aldrich	S2002
Cysteamine (MEA)	Sigma-Aldrich	30070
Glucose-oxidase	Sigma-Aldrich	G2133
Catalase	Sigma-Aldrich	C40
Acryloyl-X-SE	Thermo Fisher Scientific	A20770
Acrylamide 40%	Sigma-Aldrich	A4058
N,N′-Methylenebisacrylamide	Sigma-Aldrich	M1533
Sodium acrylate	Sigma-Aldrich	408220
TEMED	Bio-Rad	1610800
APS	Sigma-Aldrich	A3678
anhydrous-DMSO	Thermo Fisher Scientific	D12345
Guanidine HCl	Sigma-Aldrich	G3272
Triton-X-100	Sigma-Aldrich	93433
Proteinase K	Thermo Fisher Scientific	EO0492
0.1% (w/v) poly-L-lysine	Sigma-Aldrich	P8920

**Deposited data**		

Proteomics data P180 pulldown	This study	PXD050948
RNA-sequencing data P180	This study	GSE262262

**Experimental models: Cell lines**		

HEK-293T	ATCC	CRL-3216

**Experimental models: Organisms/strains**		

Rat (Wistar)	Janvier	N/A

**Recombinant DNA**		

pSuper	Brummelkamp et al.^74^	N/A
pGW1-mCherry	Kapitein et al.^75^	N/A
pGW1-BFP	Kapitein et al.^75^	N/A
RTN4A-GFP	Kind gift from dr. Voeltz (Howard Hughes Medical Institute & Department of Molecular, Cellular and Developmental Biology, University of Colorado)	Addgene #61807
pEGFP(A206K)-N1	Kind gift from dr. Lippincott-Schwartz- (Howard Hughes Medical Institute, Janelia Research Campus)	N/A
pEGFP(A206K)-C1	Kind gift from dr. Lippincott-Schwartz (Howard Hughes Medical Institute, Janelia Research Campus)	N/A
GFP-Sec61β	Kind gift from dr. Rapaport (Department of Cell Biology, Harvard Medical School)	Addgene #15108
DP1-GFP	Farías et al.^12^	N/A
RpL10A-tagRFP	Kind gift from dr. Singer (Department of Cell Biology, Albert Einstein College of Medicine)	Addgene #74172
TOM20-V5-FKBP-AP	Kind gift from dr. Ting (Department of Genetics& Biology, Stanford University)	Addgene #120914
mCherry^myr^5’/3’-Calreticulin	Kind gift from dr. Twiss (Department Biological Sciences, University of South Carolina)	N/A
Lifeact-mCherry	Kind gift from dr. Sato (Graduate School of Arts and Sciences, University of Tokyo)	Addgene #67302
HA-KifC1-MD-Strep	Farías et al.^12^	N/A
GFP-SBP-RTN4A	Farías et al.^12^	N/A
P180-ΔCoiled-coil-GFP	Farías et al.^12^	N/A
P180-ΔCoiled-coil-GFPAviTag	This study	N/A
P180-Δrepeats-GFP	Farías et al.^12^	N/A
P180-Δrepeats-GFPAviTag	This study	N/A
P180-Coiled-coil-GFPAviTag	This study	N/A
P180-Repeats-GFPAviTag	This study	N/A
SplitAP-V5-C1	Özkan et al.^19^	N/A
3xHA-split-EX-N1	Özkan et al.^19^	N/A
GFP-LRRC59	This study	N/A
RPN1-GFP	This study	N/A
KTN1-mNG	This study	N/A
P180-RCC-GFP	This study	N/A
P180-RCC-GFPAviTag	This study	N/A
Split-AP-V5-Sec61β	This study	N/A
RpL10A-3xHA-Split-EX	This study	N/A
P180-V5-Split-AP	This study	N/A
Split-AP-V5-RTN4	This study	N/A
pSuper-rat RTN4: shRNA targeting sequence: gtccagatttctctaatta	Farías et al.^12^	N/A
pSuper-rat DP1: shRNA targeting sequence: gacatataaagttccagaa	Farías et al.^12^	N/A
pSuper-rat P180-1: shRNA targeting sequence: tcagtgcaattgtctgtat	Özkan et al.^19^	N/A
pSuper-rat P180-2: shRNA targeting sequence: taaaccaaccaacacagcg	Özkan et al.^19^	N/A

**Software and algorithms**		

Fiji/ImageJ2	v2.14/1.54f	https://fiji.sc; RRID: SCR_002285
R with R Studio	v4.2.2	https://www.r-project.org
GraphPad Prism	GraphPad; v10	https://www.graphpad.com

### Resource availability

#### Lead contact

Further information and requests for resources, plasmids and reagents should be directed to and will be fulfilled by the lead contact Ginny G. Farías (g.c.fariasgaldames@uu.nl).

#### Materials availability

Plasmids in this study will be available upon request as of the date of publication.

#### Data and code availability

The mass spectrometry proteomics data generated in this study have been deposited to the ProteomeXchange Consortium via PRIDE with dataset identifier PXD050948 and analyzed data is available in Table S1. The RNA-sequencing data generated in this study has been deposited to the GEO database with identifier GSE262262 and read counts and analyzed data is available in Table S2. Raw data of all quantifications and full uncropped blots are available in Table S3. Any other data reported in this paper will be shared by the lead contact upon request.

### Experimental model and study participant details

#### Animals

All experiments were approved by the DEC Dutch Animal Experiments Committee (Dier Experimenten Commissie; AVD10800202216383), performed in line with institutional guidelines of University Utrecht, and conducted in agreement with Dutch law (Wet op de Dierproeven, 1996) and European regulations (Directive 2010/63/EU). Time-mated female pregnant Wistar rats (outbred RjHan:WI strain) were ordered from Janvier, and embryos (both genders) at E18 stage of development were used for primary cultures of hippocampal neurons. Brains from these female rats were used to obtain protein extracts. At Janvier, rats were housed in a temperature and humidity-controlled facility at a 12h/12h light cycle in cages with spruce bedding with enrichment. Pregnancy was monitored by visual examination and weighing where a significant weight gain meant gestation. Pregnant females were then shipped to the Central Laboratory Animal Research Facility of Utrecht University where they were housed for 4 days in pairs under standard laboratory conditions and received food and water ad libitum. Pregnant females were then euthanized, and embryos (E18) were harvested for primary neuronal cultures. The animals, pregnant females and embryos have not been involved in previous procedures.

#### Primary neuronal cultures and transfection

Primary hippocampal neurons were prepared from embryonic day 18 rat brains from which the hippocampi were dissected, dissociated in trypsin for 15 min at 37°C and plated at a density of 100,000/well or 50,000/well (12-well plates) on coverslips coated with poly-L-lysine (37.5 μg/mL; Sigma) and laminin (1.25 μg/mL; Roche). For experiments in developing neurons (until DIV7), neurons were maintained in neurobasal medium (NB; Gibco) supplemented with 1x B27 (Gibco), 0.5 mM glutamine (Gibco), 15.6 μM glutamate (Sigma), and 1% penicillin/streptomycin (Gibco) and incubated under controlled temperature and CO2 conditions (37°C, 5% CO2). For experiments in mature neurons (until DIV18-21) medium was refreshed weekly, starting from DIV1, by replacing half of the medium with BrainPhys neuronal medium supplemented with 2% Neurocult SM1 neuronal supplement and 1% pen/strep.

Hippocampal neurons were transfected at day in vitro (DIV)3-4 (for DIV7 experiments) or DIV10 (for DIV18-21 experiments) using Lipofectamine 2000 (Invitrogen). Shortly, DNA (0.05-2 μg/well) was mixed with Lipofectamine 2000 (1.2 μL) in Opti-MEM (Gibco, 200 μL) and incubated for 20 min at room temperature. The mix was added to the neurons in NB without additives and incubated for 1 hour at 37°C in 5% CO2. Neurons were then washed with NB 3 times and transferred back to their original medium at 37°C in 5% CO2 until fixation at DIV7.

The development of these rat primary hippocampal neurons has been well documented.^12^^,^^15^ 36-48 hours after plating (DIV3-4) one neurite will grow at least 2-3 times longer than other neurites. Axons are thus easily identifiable at DIV7 by using a fluorescent cytosolic protein fill (i.e. GFP, mCherry or BFP) since axons are on average 615 (±133) microns long whereas dendrites are much shorter (average 50-60 microns long). We additionally used an axon initial segment marker (Trim46)^14^ in some experiments to identify the axon.

#### HEK293T cell culture and transfection

Human embryonic kidney (HEK293T) cells (ATCC) were cultured in DMEM high glucose medium (Capricorn Scientific) supplemented with 10% fetal bovine serum (Gibco) and penicillin/streptomycin (Gibco). The cells were maintained at 37 °C in 5% CO2. HEK293T cells were plated into 10cm dishes (for mass spectrometry analysis) or in 60mm dishes (for pulldown-WB) and transfected using PEI MAX with different plasmids. Briefly, GFPbio constructs (varying amounts depending on the construct) together with a BirA plasmid (1:2.5 ratio) were mixed with PEI at a 1:2.5 ratio in Opti-MEM and incubated for 20 min at room temperature. Fresh DMEM medium with supplements was added to this mix and this was subsequently added to the cells which were placed back at 37 °C in 5% CO2. After 24-48 hours, cells were processed for biotin-GFP pulldowns or immunocytochemistry as described below.

### Method details

#### DNA and shRNA Constructs

Following vectors were used: pSuper,^74^ pGW1-mCherry and pGW1-BFP,^75^ RTN4A-GFP (a gift from Dr. Gia Voeltz, Addgene plasmid #61807), pEGFP(A206K)-N1 and pEGFP(A206K)-C1 (a gift from Dr. Jennifer Lippincott-Schwartz), GFP-Sec61β (a gift from Dr. Tom Rapoport, Addgene # 15108), RpL10A-tagRFP (a gift from dr. Robert Singer, Addgene #74172), TOM20-V5-FKBP-AP (a gift from Alice Ting, Addgene#120914), mCherry^myr^5’/3’-Calreticulin (a gift from dr. Jefferey L. Twiss), Lifeact-mCherry (a gift from dr. Moritoshi Sato, Addgene #67302), HA-KifC1-MD-Strep, DP1-GFP, GFP-SBP-RTN4A, P180-ΔCoiled-coil, P180-Δrepeats-GFP, P180-Coiled-coil and P180-Repeats were previously described.^12^ SplitAP-V5-C1 and 3xHA-split-EX-N1 were previously described.^19^

The plasmids generated in this study include:

For GFP-LRRC59, LRRC59 sequence was PCR amplified from a cDNA library generated from a rat INS-1 cell line and inserted in pEGFP(A206K)-C1 vector between BglII and BamHI sites using HiFi DNA assembly (New England Biosciences). A flexible linker was added before LRRC59 by addition to the cloning primers. Primers used to generate GFP-LRRC59 constructs were as follows:

5’-gctgtacaagtccggactcagcggcagcggtagcaccaagaccggtagcaagg-3’ and

5’-tcagttatctagatccggtgtcactgctgggagtcggtc-3’

For RPN1-GFP, RPN1 sequence was PCR amplified from a rat INS-1 cDNA library and inserted in pEGFP(A206K)-N1 vector between HindIII and AgeI sites by HiFi DNA assembly. A Kozak sequence was generated by addition to the cloning primers and was introduced before the RPN1 sequence. A flexible linker was inserted between RPN1 and GFP by addition to the cloning primers. The primers used to generate RPN1-GFP were:

5’- ggactcagatctcgagctcagccaccatggaggcgccgatcgtc-3’ and

5’- cttgctcaccatggtggcgacgccgcttccggatcccagagcgtccaggatgtgg-3’.

For KTN1-mNG, human KTN1 was PCR amplified from Clone I.M.A.G.E: 40125683 and inserted into pmNeonGreen-N1, between BamHI and XhoI restriction sites by HiFi DNA assembly. To generate KTN1a, the longest KTN1 isoform, DNA sequences missing from the IMAGE clone were subsequently added between aa-831 and aa-855, and between aa aa-1229 and aa-1258 by PCR.

For P180-Repeats+Coiled-coil (termed RCC) construct, the DNA sequence between aa194 and aa1540 was amplified from V5-GFP-P180 (Addgene #92150), and this fragment was cloned into pEGFP(A206K)-N1 between BamHI and XhoI sites by HiFi DNA assembly. A kozak sequence, start codon and flexible linker were inserted by addition to cloning primers. The primers used to generate this construct are:

5’- agcgctaccggactcagatcgcaccatgactggcactactcagggcaaaaag-3’ and

5’- caccatggtggcgaccggtggatccgagctaccgctgccgctacc-3’.

For GFP-AviTag versions of all P180 constructs, a GFP-AviTAG fragment was PCR amplified from GFP-AviTag-N1 and inserted into the GFP-tagged constructs between BamHI and BsrGI sites (which removes the existing GFP) by HiFi DNA assembly.

For Split APEX assay, Split-AP-V5-Sec61β, RpL10A-3xHA-Split-EX and P180-V5-Split-AP were generated.

For Split-AP-V5-Sec61β, Sec61β sequence was PCR amplified from GFP-Sec61β and inserted in Split-AP-V5-C1 vector between BglII and EcoRI. A flexible linker was introduced before Sec61β. The primers used to generate Split-AP-V5-Sec61β were:

5’-cggtagcggcagcggtagcagatctatgcctggtccgaccccc-3’ and

5’-cgcggtaccgtcgactgcagaattcctacgaacgagtgtacttgcccc-3’.

For RpL10A-3xHA-Split-EX, RpL10A sequence was PCR amplified from RpL10A-TagRFP and inserted in 3xHA-Split-EX-N1 vector between AgeI and HindIII. A flexible linker was introduced after RpL10A. The primers used to generate RpL10A-3xHA-Split-EX were:

5’-ggactcagatctcgagctcaagcttgccaccatgagcagcaaag-3’ and

5-caggaacatcgtatgggtaaccggtgctaccgcttccggatccatacagacg-3’.

For P180-V5-Split-AP, V5-Split-AP was PCR amplified from TOM20-V5-AP and inserted in P180-FL-GFP vector between BsrGI and BamHI sites (which removes existing GFP). A flexible linker was added after P180 sequence. The primers used to generate P180-V5-Split-AP were:

5’-gtagcggcagcggtagcccggatccggatccaggtaagcctatccctaacc-3’ and

5’-gagtcgcggccgctttacttgtacatgtacattaggcatcagcaaacccaag-3’.

For Split-AP-V5-RTN4, RTN4 sequence was PCR amplified from GFP-SBP-RTN4 and inserted into Split-AP-V5-C1 vector between BglII and EcoRI restriction sites. A flexible linker was introduced before RTN4. The primers used to generate Split-AP-V5-RTN4 were:

5’-cggtagcggcagcggtagcagatctatggaagacctggaccag-3’ and

5-cgcggtaccgtcgactgcagaattctcattcagctttgcgcttc-3’.

The following sequences for rat-shRNAs were used in this study: RTN4-shRNA (5’-gtccagatttctctaatta-3’), DP1-shRNA (5’-gacatataaagttccagaa-3’) validated in Farías et al.^12^; P180-shRNAs (5’-tcagtgcaattgtctgtat-3’ and 5’-taaaccaaccaacacagcg-3’) validated in Özkan et al.^19^

#### Antibodies and reagents

The following primary antibodies were used in this study: mouse anti-Puromycin (Kerafast Cat# EQ0001, RRID:AB_2620162, 1:3000), rabbit anti-RpS12 (Proteintech Cat#16490-1-AP, RRID:AB_2146233, 1:200 for IC, 1:1000 for WB), rabbit anti-RpL24 (Proteintech Cat# 17082-1-AP, RRID:AB_2181728, 1:200 for IC, 1:1000 for WB), rabbit-anti-Trim46 (homemade, van Beuningen et al.,^14^ 1:700 for IC), mouse anti-V5 (Thermo Fisher Scientific Cat# R960-25, RRID:AB_2556564, 1:1000 for IC), rat anti-HA (Roche Cat# 11867423001, RRID:AB_390918, 1:1000 for IC), mouse-anti-GFP (Thermo Fisher Scientific Cat#A-11120; RRID:AB_221568, 1:250-500 for IC), rabbit-anti-GFP (Abcam Cat#ab289; RRID:AB_303395, 1:10.000 for WB).

The following secondary antibodies were used in this study: Alexa-Fluor555 conjugated Strep (Thermo Fisher Scientific Cat# s21381, RRID:AB_2307336, 1:2000), Alexa-Fluor-568 conjugated Strep (Thermo Fisher Scientific Cat# S-11226, RRID:AB_2315774, 1:1000), goat anti-Mouse IgG (H+L) Highly Cross-Adsorbed Alexa Fluor 488 (Thermo Fisher Scientific Cat# A-11029, RRID:AB_2534088, 1:1000 for IC, 1:200 for TREx), goat anti-mouse Alexa568 (Thermo Fisher; Cat#A-11031, RRID:AB_144696, 1:1000), goat anti-mouse Alexa405 (Molecular Probes, Cat# A31553, RRID:AB_221604, 1:500), goat anti-rat Alexa 488 (Thermo Fisher Scientific Cat# A-11006, RRID:AB_2534074, 1:1000). Anti-rabbit IRDye 800CW (Li-Cor, Cat#926-32211, RRID:AB_621848), Atto488 conjugated Fluotag-X4® GFP nanobody (NanoTag Biotechnologies, Cat# N0304-At488-L, 1:250), goat anti-rabbit IgG (H+L) Highly Cross-Adsorbed CF®594 (Sigma-Aldrich, Cat#SAB4600110, 1:1000), goat anti-mouse IgG Abberior STAR 635P(Abberior Cat# ST635P-1001-500UG, RRID:AB_2893232, 1:500), goat anti-rabbit Alexa 647 (Thermo Fisher Scientific Cat# A-21245, RRID:AB_2535813, 1:500), goat anti-mouse CF680 (Biotium Cat# 20065, RRID: AB_10557108, 1:500), anti-rabbit ATTO 647N (Sigma-Aldrich, Cat#40839, RRID:AB_1137669, 1:200).

Other reagents used in this study were: Puromycin dihydrochloride (Sigma-Aldrich, Cat# P8833), Anisomycin (Sigma-Aldrich, Cat#9789), recombinant human Neurotrophin-3 (NT-3) protein (50ng/ml, Alomone labs, Cat#N-260), recombinant human BDNF protein (50ng/ml, Alomone labs, Cat#B-250), recombinant rat beta-NGF Protein (50ng/ml, R&D systems, Cat# 556-NG). NeutrAvidin (Thermo Fisher Scientific, Cat# 31000), Heme (Sigma-Aldrich, Cat# 51280); biotin-phenol (Iris Biotech, Cat#LS.3500); H2O2 (Sigma-Aldrich, Cat#H1009), Lipofectamine 2000 (ThermoFisher Scientific, Cat#11668019), Polyethylenimine (PEI MAX; Polysciences, Cat#24765), Fluoromount-G Mounting Medium (ThermoFisher Scientific, Cat#00-4958-02), Dynabeads Streptavidin (Thermo Fisher, Cat#11205D), Pierce Streptavidin Magnetic beads (Thermo Fisher, Cat#88816), RNase A (Thermo Fisher, Cat#EN0531), RNaseT1 (Thermo Fisher, Cat#EN0541), SUPERase-in RNase Inhibitor (Thermo Fisher, Cat# AM2694), Trolox (Sigma-Aldrich, Cat#238831), Sodium L-Ascorbate (Sigma-Aldrich, Cat#A4034), Sodium Azide (Sigma-Aldrich, Cat#S2002), Cysteamine (MEA) (Sigma, Cat#30070), Glucose-oxidase (Sigma, Cat#G2133), Catalase (Sigma, Cat#C40), Acryloyl-X-SE (AcX, Thermo Fisher Scientific, Cat# A20770), Acrylamide (40%, Sigma, Cat#A4058-100ML), N,N′-Methylenebisacrylamide (bisacrylamide, Sigma, Cat#M1533-25ML), Sodium acrylate (Sigma, Cat# 408220-25g), TEMED (Bio-Rad, Cat#1610800), APS (Sigma, Cat#A3678), anhydrous-DMSO (Thermo-Fisher Scientific, Cat# D12345), Guanidine HCl (Sigma, Cat#G3272), Triton-X-100 (Sigma, Cat#93433), Proteinase K (Thermo Fisher Scientific, Cat#EO0492) and 0.1% (w/v) poly-L-lysine (Sigma, Cat#P8920-100mL).

#### Puromycilation assay

In order to label newly synthesized proteins, we briefly incubated neurons with 10μM puromycin for 10 minutes. This timeframe has commonly been used to assay newly synthesized proteins in axons^21^^,^^23^ or dendrites.^22^ After 10 minutes, unincorporated puromycin was washed out using two washes with NB medium and neurons were fixed immediately and processed for immunostaining as described below. For control experiments, neurons were pre-treated with 40 μM anisomycin for 30 minutes or puromycin was omitted.

#### Immunofluorescence staining and confocal imaging

Neurons were fixed with pre-warmed 4% paraformaldehyde plus 4% sucrose in PBS for 10-20 min at RT and washed with PBS supplemented with calcium and magnesium (PBS-CM) three times. Fixed cells were subsequently permeabilized with 0.2% Triton X-100 in PBS-CM for 15 min at RT, washed 3 times and were then incubated with blocking buffer (0.2% porcine gelatin in PBS-CM) for 1 hour at RT. Next, neurons were incubated with primary (1 hour at RT or O/N at 4°C) and secondary antibodies (1h at RT) at specified concentrations in blocking buffer. Coverslips were mounted in Fluoromount-G Mounting Medium and imaged by using a confocal laser-scanning microscope (LSM700, with Zen imaging software (Zeiss) version 8.1.7.484) equipped with Plan-Apochromat ×63 NA 1.40 oil DIC and EC Plan-Neofluar x40 NA1.30 Oil DIC objectives or processed and imaged with super-resolution microscopy as described below.

#### Stimulated Emission Depletion (STED) microscopy

Imaging was performed with a Leica TCS SP8 STED 3x microscope using an HC PL APO 100x/NA 1.4 oil immersion STED WHITE objective. The 488, 561 and 633 nm wavelengths of pulsed white laser (80MHz) were used to excite Atto488, CF®594 and Star635P, respectively. To obtain gSTED images, Atto488 was depleted with the 592 nm continuous wave depletion laser, CF®594 and Star635P were depleted with the 775 nm pulsed depletion laser. An internal Leica HyD hybrid detector with a time gate of 0.3 to 6 ns was used. Images were acquired as Z-stack and maximum intensity projections were obtained for image display and analysis.

#### Ten-fold Robust Expansion (TREx) microscopy

Ten-fold robust expansion microscopy was performed as previously described^29^ with slight alterations. Briefly, neurons were fixed and immunostained as described above (using a two-fold higher concentration for primary antibodies) after which they were anchored with 100ug/ml acryloyl-X SE in PBS overnight at RT. After rinsing in PBS, coverslips containing the cells were inverted cell-side down onto pre-cooled gelation chambers (using silicon rings (13mm diameter, 120μL volume, Sigma, Cat# GBL664107)), containing the gelation solution (1.08 M sodium acrylate, 14.4% (v/v) acrylamide, 0.009% (v/v) bis-acrylamide, 1.5% TEMED, 0.15% (w/v) APS in PBS) on ice. Gelation occurred at 37°C for 30 min. Samples were subsequently rinsed in PBS and submerged in digestion buffer (0.5% Triton-X-100, 0.8M Guanidine-HCl, 9U/mL Proteinase K in TAE buffer (40mM Tris, 20mM acetic acid, 1mM EDTA) and digested for 4h at 37°C, followed by brief rinsing with MilliQ (MQ) water. Samples were cut into smaller gel pieces to allow for faster expansion and transferred into 15cm cell culture dishes filled with MQ to promote physical sample expansion through osmosis (fresh MQ was exchanged three times over a course of 2 days to allow for complete sample expansion with an estimated expansion factor of ∼9 calculated using the overall increase in size of the gel measured before mounting and imaging the sample, compared to the initial gel size after gelation). Samples were subsequently cut with a razor and mounted on 3D printed sample holders (printed with PLA using a Prusa mini printer and a model generated in Fusion360, see.obj file) onto a poly-L-lysine coated, and plasma-cleaned, rectangular coverslip.

Imaging was performed on a Leica SP8 3X STED microscope using a 63x (HC PL APO CS2, NA1.20) water objective using confocal setup. Detailed acquisition settings were the following: bidirectional scanning, 400Hz speed, zoom factor 7, 1AU pinhole, twofold frame averaging, 30% laser intensity (at 488nm, 8-fold line accumulation, detection using a HyD at 495nm - 570nm and gating set to 0.3ns – 6ns) and 30% laser intensity (at 633nm, three-fold line accumulation, detection using a HyD at 644nm - 736nm and gating set to 0.3ns – 6ns). Images were acquired using the sequential image settings as a z-stack over a physical distance of 0.92nm using three slices to capture the axon.

#### Dual-color Single Molecule Localization Microscopy (SMLM)

Dual-color SMLM was performed as previously described.^30^ Briefly, we used a method similar to spectral demixing, to classify two spectrally very close far-red fluorophores enabling high-resolution stochastic optical reconstruction microscopy (STORM) imaging of two channels. Neurons were fixed and immunostained as described above using goat anti-rabbit AlexaFluor (AF) 647 and goat anti-mouse CF680 secondaries. Samples were mounted in imaging buffer (100mM MEA, 5% w/v glucose, 700 μg/ml glucose oxidase, 40 μg/ml catalase in 50mM Tris pH 8.0) in closed off cavity slides (Sigma, BR475505) to prevent oxygen from entering the sample during imaging. Imaging was performed on a Nikon TI-E microscope equipped with a TIRF APO x100 NA 1.49 oil objective lens. A 638 nm laser (MM, 500mW, Omicron) together with a laser clean-up filter (LL01-638, Semrock) and excitation dichroic (FF649-Di01, Semrock) was used to excite the sample. The collected emission was relayed through an Optosplit III module (Cairn Research), fitted with emission dichroic (FF660-Di02) to split the emission in a short channel and a long channel on a EMCCD (iXon 897 – Andor). Samples were imaged with laser at a TIRF angle, for 16000 frames with an exposure of 10 ms. Acquisitions were analyzed using the custom ImageJ plugin DoM (Detection of Molecules, https://github.com/ekatrukha/DoM_Utrecht), and reconstructions were generated by plotting the resulting localizations. Further analysis was performed using PFC (Probability-based Fluorophore Classification).^30^ Using the short channel for classification with a generalized likelihood ratio test (GLRT) and the long channel for localization, fluorophores AF647 and CF680 are separated. The result is two high resolution reconstructions of our proteins of interest. For Figure 2K, neurons were treated with a high concentration (200μM) of puromycin for 45 minutes before fixation and processing as described above.

#### Live-cell imaging of axon growth

Live-cell imaging of growth cones were performed in an inverted microscope Nikon Eclipse Ti-E (Nikon), equipped with a Plan Apo VC 100x NA 1.40 oil and a Plan Apo VC 60x NA 1.40 oil objective (Nikon), a Yokogawa CSU-X1-A1 spinning disk confocal unit (Roper Scientific), a Photometrics Evolve 512 EMCCD camera (Roper Scientific) and an incubation chamber (Tokai Hit) mounted on a motorized XYZ stage (Applied Scientific Instrumentation) which were all controlled using MetaMorph (Molecular Devices) software. Coverslips were mounted in Ludin chambers and imaged using an incubation chamber that maintains temperature and CO2 optimal for the cells (37°C and 5% CO2). Imaging was performed in the original full conditioned medium from neurons in culture. For dual-color videos, different laser channels used to visualize fluorescently tagged proteins were sequentially exposed for 100-250 ms. Total time and intervals of imaging acquisition for each experiment are indicated in each legend for Figure and/or legend for Movie. Neurons expressing Lifeact-mCherry together with GFP or RCC-GFP were imaged at DIV3-4. Images were acquired at 1 min interval for Lifeact-mCherry, and only the first and last frames were acquired for GFP or RCC-GFP to avoid phototoxicity.

#### Fluorescence recovery after photobleaching

For mCherry^myr^5’/3’-Calreticulin fluorescence recovery after photobleaching, the experiments were performed in neurons DIV4 using the ILas2 system (Roper Scientific). A region of interest of 40 x 40 μm was chosen at the most distal axon. Bleaching was performed with 80 repeats, using 100% laser power after 30s of recording, and fluorescence recovery was analyzed for a period of 15 min with imaging acquisition every 30s. To determine if fluorescence recovery in axons was from translation, cultures were treated with 100 μM Anisomycin (Sigma) for 45 min prior to photobleaching.

#### Streptavidin/SBP heterodimerization system assay

Controlled coupling of ER tubules to MT-driven motor proteins has been previously described.^12^ Briefly, neurons were transfected at DIV4 with only GFP-SBP-RTN4A as a control or GFP-SBP-RTN4A plus HA-KifC1-MD-Strep to pull axonal ER tubules to soma. NeutrAvidin (0.3 mg/ml) was added to the neurons after 1 h of transfection to prevent Strep-SBP uncoupling.

#### Split APEX assay

Split APEX assay was performed as described previously.^19^ Briefly, neurons were transfected at DIV4 with RpL10A-HA-EX and AP-V5-Sec61β, V5-AP-RTN4A or P180-V5-AP constructs. At DIV7, neurons first were incubated with heme (6 μM for 60 min at 37°C/ 5% CO2 and subsequently washed once with NB and incubated with biotin-phenol (500 μM) in fresh NB with supplements for 30 min at 37°C/ 5% CO2. For cue stimulation experiments, cues were added together with biotin phenol for 30 min at 50ng/ml. Then, H2O2 to a final concentration of 1 mM was introduced to the medium for 1-2 min to initiate proximity labelling, after which the reaction was stopped by removing the medium and washing the cells once with quenching buffer (5 mM Trolox and 10 mM sodium ascorbate in HBSS) containing 10 mM sodium azide and twice with quenching buffer without sodium azide for 3–5 min each at 37°C/ 5% CO2. Neurons were subsequently fixed and immunostained as described above.

#### Biotin-GFP pulldown assays

Dynabeads-M280-Streptavidin or Pierce Streptavidin magnetic beads were first blocked with a blocking buffer (20 mM Tris HCl pH7.5, 150 mM KCl and 0.2 ug/ul chicken egg albumin) for 1 hour by rotating at RT at 16 rpm and were then washed with wash buffer (20 mM Tris HCl pH7.5, 150 mM NaCl, 0.1% Triton-X 100 and 5 mM MgCl2) for 3 times using a magnetic rack.

Cells were collected by first washing them with ice-cold 1x PBS supplemented with 0.5x protease inhibitor cocktail (Roche) and then collecting them with a cell scraper in PBS/0.5x protease inhibitor in an eppendorf tube. Cells were pelleted by centrifugation at 3000g for 5 min at 4 °C. Cell pellets were resuspended in lysis buffer (20 mM Tris HCl pH7.5, 100 mM NaCl, 1% Triton-X 100, 5 mM MgCl2 and 1x protease inhibitor cocktail and then subsequently incubated at 4 °C by rotating at 16 rpm for 15-30 min. Lysed cells were cleared by centrifugation at 4 °C, 16100g for 20 min. 5% of the total lysate was kept as input sample and the remaining lysate was added to streptavidin beads and incubated at 4°C by rotating at 16 rpm for 1 hour. After this, beads were washed 4 times with 400ul wash buffer on a magnetic rack. For RNAse A/T1 treament, RNAse A (4ng/μl) and RNaseT1 (250U/ml) were added to wash buffer and beads were wash 4 times on a magnetic rack. Finally, beads were dissolved in 2x Leammli sample buffer with DTT and boiled at 95°C for 10 min and the sample was then separated from the beads on a magnetic rack and transferred to a new tube. Samples were stored at –20°C until processing for mass spectrometry or Western blot. For pulldowns using rat brain lysates, beads incubated with HEK293T lysates as above were washed twice with low salt buffer (100mM KCl, 0.1% TritonX-100, 20mM Tris, pH 7.6), twice with high salt buffer (500mM KCl, 0.1% TritonX-100, 20mM Tris, pH 7.6) and twice again with low salt buffer to remove binding proteins from HEK293T cells. Beads were then incubated with whole rat brain extract for 1h at 4°C and subsequently washed 5 times using normal wash buffer and sample was then collected from the beads as above.

For RNA-immunoprecipitation, the same procedure was followed with a few adaptations. SUPERase-in RNase Inhibitor (Thermo Fisher) was added to lysis and wash buffers. After bead incubation and 4 washes, RLT buffer (QIAGEN) + β-mercaptoethanol was added to the beads and incubated with vortexing for 2 minutes. The sample was then separated from the beads on a magnetic rack and transferred to a RNeasy column for RNA isolation (QIAGEN) according to protocol. RNA was stored at -80°C until processing for RNA-sequencing.

#### Western blot analysis

Samples were loaded on a home-made 9 or 10% Bis-Acrylamide (Bio-Rad) gel and the gel was subsequently transferred by wet transfer to a PVDF membrane (Bio-Rad) for 90 min at 100V. The blots were blocked in blocking buffer (5% skimmed milk in TBS-T) for 1 h at room temperature. Blots were then incubated with primary antibodies diluted in blocking buffer at desired concentrations overnight at 4°C on a rotator. Blots were washed with TBS-T 3 times for 5 min each on a shaker and incubated with secondary antibody (Li-Cor) in blocking buffer at desired concentrations for 1 h at room temperature in the dark. Finally, blots were washed 3 times with TBS-T for 5 min each and twice briefly in TBS before developing on an Odyssey CLx imaging system (Li-Cor) with Image Studio version 5.2 software. Protein levels were analyzed and normalized by importing images from Western blot detection into Fiji/ImageJ. SDS-PAGE silver stain was performed using a Pierce Silver Stain Kit (Thermo Fisher; Cat#24612).

#### Mass Spectrometry sample preparation and analysis

For mass spectrometry analysis, pulldown samples collected as described above were loaded on a 4-12% gradient Criterion XT Bis-Tris precast gel (Bio-Rad). The gel was fixed in 40% methanol and 10% acetic acid and subsequently stained for 1h using colloidal coomassie dye G-250 (Gel Code Blue Stain Reagent, Thermo Fisher). Each lane from the gel was cut into 3 gel pieces and placed in 0.5-mL tubes. The gel pieces were washed with water, followed by 15 min dehydration in acetonitrile. Proteins were reduced (10mM DTT for 1h at 56°C), dehydrated and alkylated (55 mM iodoacetamide for 1h in the dark). After two rounds of dehydration, digestion was performed by adding trypsin (Promega; 20μl of 0.1 mg/ml in 50 mM ammonium bicarbonate) and incubating overnight at 37°C. Peptides were extracted with acetonitrile, dried down and reconstituted in 10% formic acid.

All samples were analyzed on an Orbitrap Q-Exactive mass spectrometer (Thermo Fisher) coupled to an Agilent 1290 Infinity LC (Agilent Technologies). Peptides were loaded onto a trap column (Reprosil C18, 3μm, 2cm x 100μm) with solvent A (0.1% formic acid in water) at a maximum pressure of 800 bar and chromotographically separated over the analytical column (Zorbax SB-C18, 1.8μm, 40cm x50μm; Agilent) using 90 min linear gradient from 7-30% solvent B (0.1% formic acid in acetonitrile) at a flow rate of 150 nL/min. The mass spectrometer was used in a data-dependent mode, which automatically switched between MS and MS/MS. After a survey scan from 350-1500 m/z the 10 most abundant peptides were subjected to HCD fragmentation.

For data analysis, raw files were processed using Proteome Discoverer 1.4 (v1.4.14, Thermo Fisher). Database searches were performed using Mascot as a search engine (v2.5.1, Matrix Science) on the Human and Rat Uniprot databases. Carbamidomethylation of cysteines and oxidation of methionine were set as fixed and variable modifications respectively. Trypsin was set as cleavage specificity, allowing a maximum of 2 missed cleavages. Data filtering was performed using percolator, resulting in 1% false discovery rate. Additional filters were ‘search engine rank 1’ and ‘mascot ion score >20’. To infer protein abundance of each protein pulled down with the bait protein, we relied on total numbers of peptide spectrum matches (PSM). Dot plots of PSM and peptide numbers for selected proteins were generated using a custom-made script in R (R-project).

#### RNA-sequencing and data analysis

Sequencing was performed at Single Cell Discoveries (Utrecht, The Netherlands). Isolated RNA as described above was used for library preparation and sequencing. mRNA was processed as described previously, following an adapted version of the single-cell mRNA seq protocol CEL-Seq2.^76^^,^^77^ In brief, samples were barcoded with CEL-seq primers during a reverse transcription and pooled after second strand synthesis. The resulting cDNA was amplified with an overnight in vitro transcription reaction. From this amplified RNA, sequencing libraries were prepared with Illumina Truseq small RNA primers. The DNA library was paired-end sequenced on an Illumina Nextseq 500, high output, with a 1x75bp Illumina kit (R1: 26 cycles, index read: 6 cycles, R2: 60 cycles).

Read 1 was used to identify the Illumina library index and CEL-seq2 sample barcodes. Read 2 was aligned to the *Rattus norvegicus* mRatBN7 reference genome using STARSolo 2.7.10a.^78^ Sample data was demultiplexed by using je-suite-2.0.^79^ Reads that mapped equally well to multiple locations were discarded. Mapping and generation of count tables were done using the STARSolo 2.7.10a aligner.

Differential gene expression analysis was performed in R using the DeSeq2 package. Only reads with a count of 10 or higher were included for analysis. FDR was set at <0.05. GO analysis was performed using DAVID gene ontology analysis (https://david.ncifcrf.gov/). For GO analysis on axonally translated P180-enriched mRNAs, we analyzed mRNAs that were overlapping with mRNAs identified as axonally translated at age P0.5^4^ and in Jung et al.^27^

### Quantification and statistical analysis

#### Image analysis and quantification

For all analysis performed in axonal segments, the axons of DIV7 neurons were identified by either using an axon initial segment marker (Trim46)^14^ or by identifying the longest neurite based on a fluorescent protein fill (i.e. cytosolic GFP, mCherry or BFP; Figure S1C). As mentioned above, at DIV7 of our primary hippocampal neurons the axons are easily identifiable as such by using a fluorescent protein fill since they are on average 615 (±133) microns long whereas dendrites are much shorter (average 50-60 microns long).

##### Puromycilation and ribosomal protein intensity

To analyze puromycilated peptide and ribosomal protein intensities, samples were imaged with the same settings for laser power, exposure and gain for all conditions. Distal axonal segments were selected for imaging by moving along the axon using a cell fill until the axon tip was reached. The axon was identified as described above. Next, an image of an axonal segment in this distal region (400-600 microns away from soma; Figure S1C) that did not have many crossing neurites from other, untransfected neurons, was selected for imaging. Fiji/ImageJ was used to quantify the background corrected intensity. Average z-projections from acquired images were generated and segmented lines were manually drawn along axon segments of 30-50 microns (1-4 segments per neuron). The average area measured between conditions was similar. Mean background corrected intensities from 16-bit images were measured.

##### Polarity index of ER proteins

Quantification of polarized distribution of proteins/organelles in neurons, polarity index (PI) has been previously described.^75^ Shortly, Fiji/ImageJ was used to draw segmented lines along an axonal region of ∼200 μm after the axon initial segment, and three dendrites per neuron. Mean intensities in axon and dendrites were measured. The following formula was applied PI = (Id- Ia)/(Id+Ia): where Id is the average mean intensity of the three dendrites and Ia is the mean intensity of axon. Non-polarized distribution represented by PI = 0 where Id = Ia, PI<0 indicates axonal distribution and PI>0 indicates dendritic distribution.

##### Split APEX streptavidin intensity quantification

For streptavidin signal intensity analysis, samples were imaged with the same settings for laser power, exposure and gain for all conditions. Axonal segments along the axon were selected for imaging. Fiji/ImageJ was used to quantify the background corrected intensity of signals. Average z-projections of images were generated and segmented lines were manually drawn along the axon. Mean intensities from 16-bit images were measured. The intensities for the strep signal were normalized to control conditions and per experiment. For comparison of Sec61β and RTN4 split APEX, the streptavidin intensities were additionally correct for V5 and HA intensities.

##### Quantification of ribosomes bound to ER with superresolution microscopy

To quantify the portion of ribosomes in contact with the axonal ER from STED, TREx and dual-color SMLM images, axonal segments were first straightened in Fiji/ImageJ. The mean intensity of the ribosomal channel was measured first. Then, masks from the Sec61β-GFP (ER) channel were generated by thresholding this channel. An outline of this mask was created and the intensity of the ribosomal channel within this mask was measured. For 'ER enlarged' the mask was enlarged with 5nm and mean ribosomal protein intensity was measured. The proportion of ribosomal protein intensity within the mask from the total amount was then calculated and plotted.

##### FRAP analysis

The mean intensity of the bleached area was obtained and corrected with background values, as well as the bleaching that occurred during image acquisition. Data were normalized with control fluorescence averaged over 3 initial frames before bleaching and stated as 100% intensity, while the initial bleaching point is stated as 0% intensity. Average curves from multiple neurons were obtained and represented.

##### Neuron morphology analysis

To quantify neuron complexity and morphology, images containing the whole neuron were analyzed. When necessary, images were stitched together using the pairwise stitching plugin in ImageJ.^80^ Images were then tresholded based on a mCherry fill and Sholl analysis was performed using the Neuroanatomy plugin in ImageJ. The soma was manually selected as the center and the step size of each radius was set to 10μm. The intersections per radius or total intersections were used for data analysis.

##### Growth cone dynamics analysis

Kymograph from live cell imaging were generated using ImageJ, as previously described (Farías et al., 2015). Briefly, segmented lines of several thickness and length were traced along axonal tips. Kymographs were generated from straightened lines by re-slicing stacks followed by z projection. All tracks were orientated so that anterograde movement occurred from left to right. Length of segmented line, as well as time of recording are shown in each kymograph. Axonal growth cones were visualized with Lifeact-mCherry over a recording time of 90 min. Changes in actin waves were analyzed and frequencies are represented in the pie charts for GFP and RCC-GFP transfected cells.

#### Statistical analysis

Data processing and statistical analysis were performed using Microsoft Excel and GraphPad Prism. Unpaired t-tests, Mann-Whitney tests, ordinary one-way ANOVA tests followed by Dunnett's multiple comparisons tests or with two-stage Benjamini, Krieger and Yekutieli FDR procedure were performed for statistical analysis as indicated in figure legends. See Table S3 for details on number of experiments, type of analysis and statistical test per experiment.

## Supplemental information

Document S1. Figures S1–S5

Table S1. Data table containing proteomics data for GFPbio pulldowns in HEK293 cells and rat brain extracts, related to Figures 4 and S4

Table S2. RNA-sequencing analysis data table for GFPbio RNA immunoprecipitation RNA-sequencing from primary cortical neurons, related to Figure 6

Table S3. Table with the source/raw data for each quantification and uncropped blots presented in the figures and detailed experimental information including N replicates, n number, and statistical tests, related to Figures 1, 2, 3, 4, 5, 6, 7, S1, S3, and S4

Document S2. Article plus supplemental information
