# Supplementary material for: Axonal endoplasmic reticulum tubules control local translation via P180/RRBP1-mediated ribosome interactions
Source: Dev Cell. 2024 Aug 19;59(16):2053–2068.e9. doi: 10.1016/j.devcel.2024.05.005 (PMC11338522; doi:10.1016/j.devcel.2024.05.005)
Supplement: Document S1. Figures S1–S5 [file mmc1.pdf]

Developmental Cell, Volume *59*

Supplemental information

Axonal endoplasmic reticulum tubules control local translation via P180/RRBP1-mediated ribosome interactions

Max Koppers, Nazmiye Özkan, Ha H. Nguyen, Daphne Jurriens, Janine McCaughey, Dan T.M. Nguyen, Chun Hei Li, Riccardo Stucchi, Maarten Altelaar, Harold D. MacGillavry, Lukas C. Kapitein, Casper C. Hoogenraad, and Ginny G. Farías

Figure S1 (related to Figure 1)

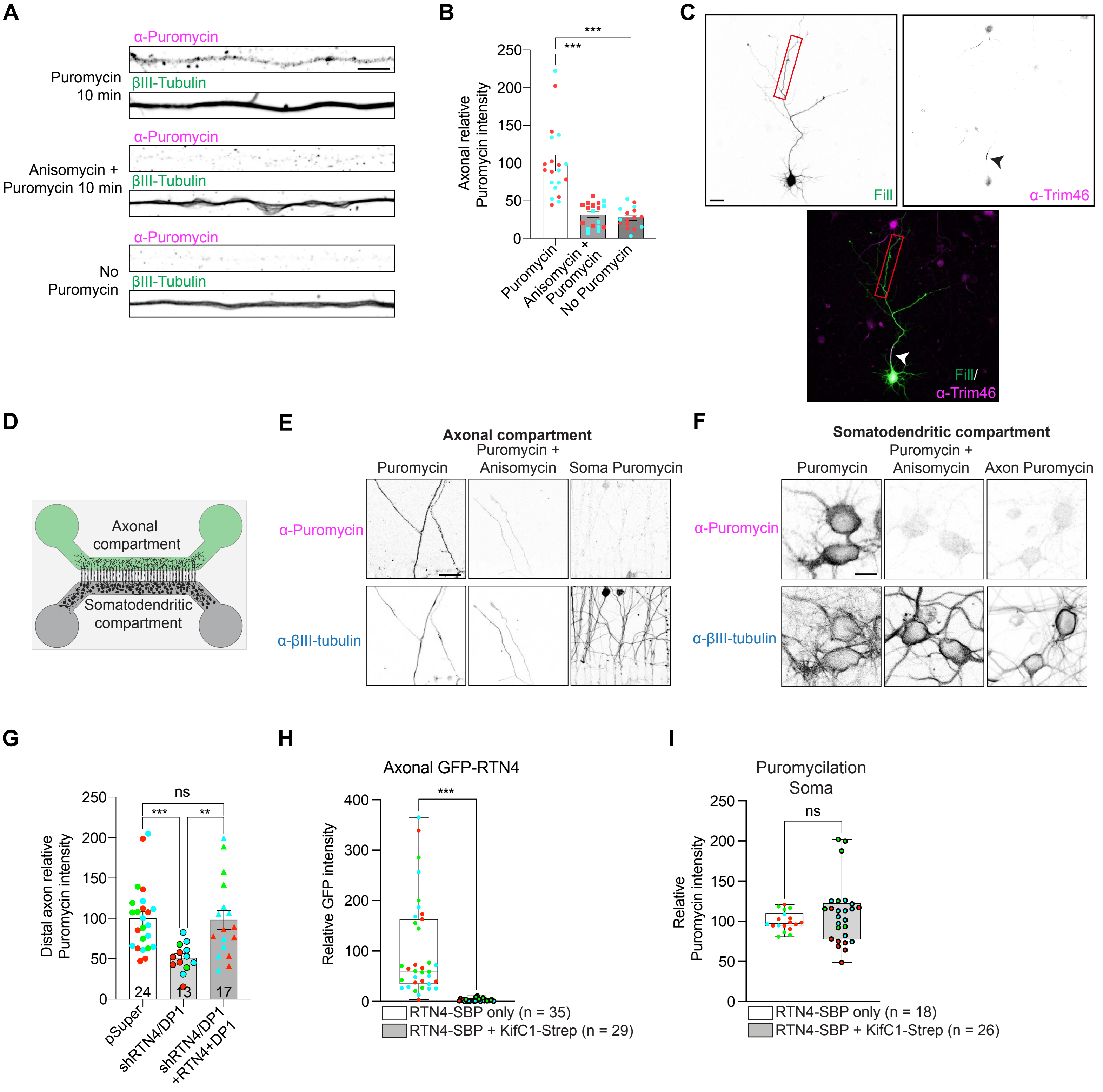

Fig. S1. (Related to Figure 1). Axonal puromycilation specificity, efficient and selective ER removal from the axon which does not affect somatic puromycilation

(**A**) Representative images of puromycilated peptides in axons from neurons treated with puromycin for 10 min (top), treated with puromycin for 10 min after anisomycin pre-treatment for 30 min (middle) or without puromycin (bottom). Scale bar represents 5 μm.

(**B**) Quantification of axonal puromycin intensity in neurons relative to puromycin treated neurons.

(**C**) Representative images of a DIV7 neuron transfected with a GFP fill and stained with the axon initial segment marker Trim46. This shows the clearly polarized nature of these neurons with a clearly formed axon. The red box marks an example of a distal axonal region as used in quantifications of Figure 1. Scale bar represents 20 μm.

(**D**) Schematic of a microfluidic chamber showing the somadendritic and axonal compartments

(**E**) Images of anti-puromycin and anti-BIII-Tubulin staining taken from the axons in the axonal compartment treated with puromycin for 10 minutes (left), treated with puromycin for 10 min after anisomycin pre-treatment for 30 min (middle) or treated with puromycin in the somatodendritic compartment (right). Scale bar represents 25 μm.

(**F**) Images of anti-puromycin and anti-BIII-Tubulin staining taken from neurons in the somatodendritic compartment treated with puromycin for 10 minutes (left), treated with puromycin for 10 min after anisomycin pre-treatment for 30 min (middle) or treated with puromycin in the axonal compartment (right). Scale bar represents 25 μm.

(**G**) Quantification of axonal puromycin intensity in distal axons of DIV7 neurons transfected with a fill and a pSuper plasmid (control), pSuper plasmids containing shRNA targeting RTN4 and DP1 or pSuper plasmids containing shRNA targeting RTN4 and DP1 together with RTN4-GFP and DP1-GFP (rescue).

(**H**) Quantification of RTN4-SBP-GFP levels in distal axons of DIV7 neurons expressing a fill plus RTN4-SBP-GFP in absence or presence of Strep-KifC1.

(**I**) Quantification of puromycin intensity in neuronal soma of DIV7 neurons expressing a fill and RTN4-SBP-GFP in absence or presence of Strep-KifC1.

Individual data points each represent a neuron and each color represents an independent experiment. Data are presented as mean values ± SEM in (**B, G, H, I**). ns = non-significant, ***$p<0.001$ comparing conditions to control using one-way ANOVA with Dunnett's multiple comparison test (B, G) or Mann-Whitney tests (H, I).

Figure S2 (related to Figure 2)

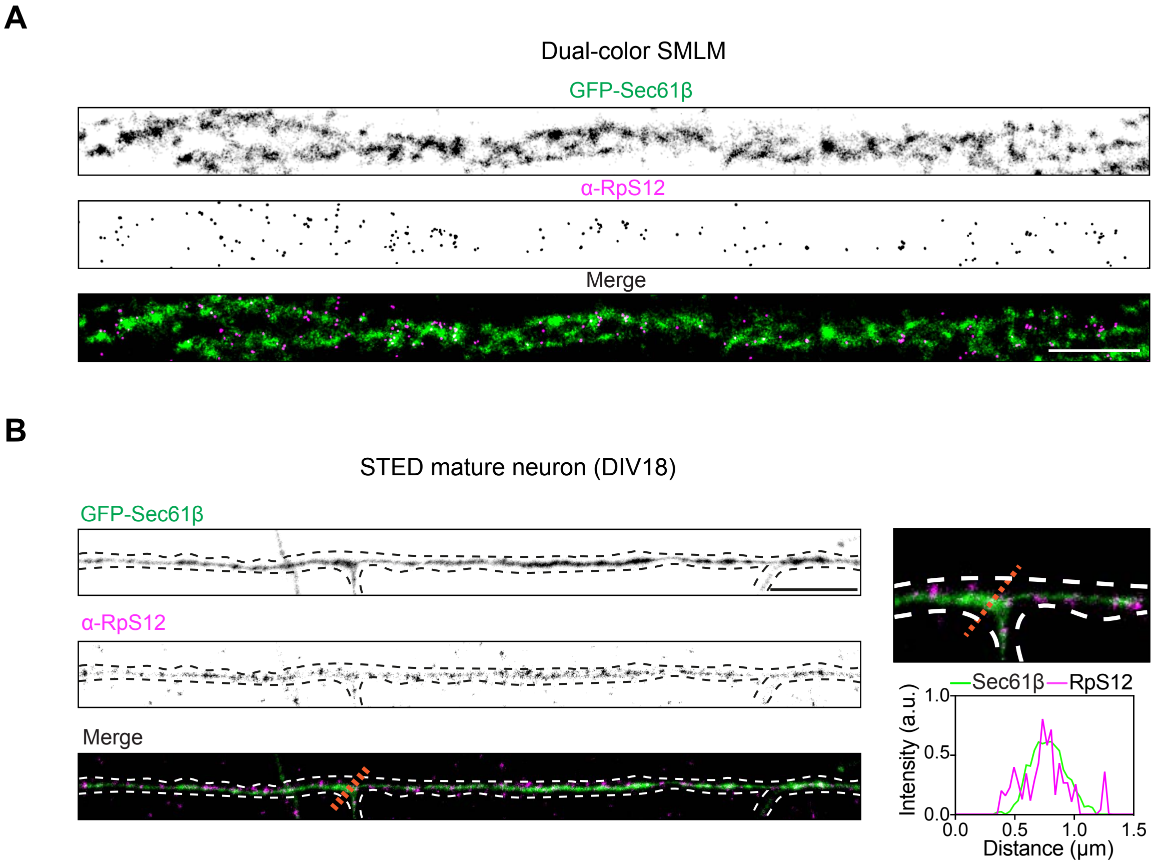

Fig. S2. (Related to Figure 2). Super-resolution imaging of axonal ER – ribosome interactions

(**A**) Representative dual-color STORM image of the ER and ribosomes in axons from neurons expressing GFP-Sec61β and stained for RpS12 of a large axonal segment.
(**B**) Representative STED image of the axon of a DIV21 mature neuron expressing GFP-Sec61β and stained for RpS12. A zoomed image and intensity profile plot are shown at the bottom.
Scale bars represent 1µm (**A**) and 5µm in (**B**).

Figure S3 (related to Figure 3)

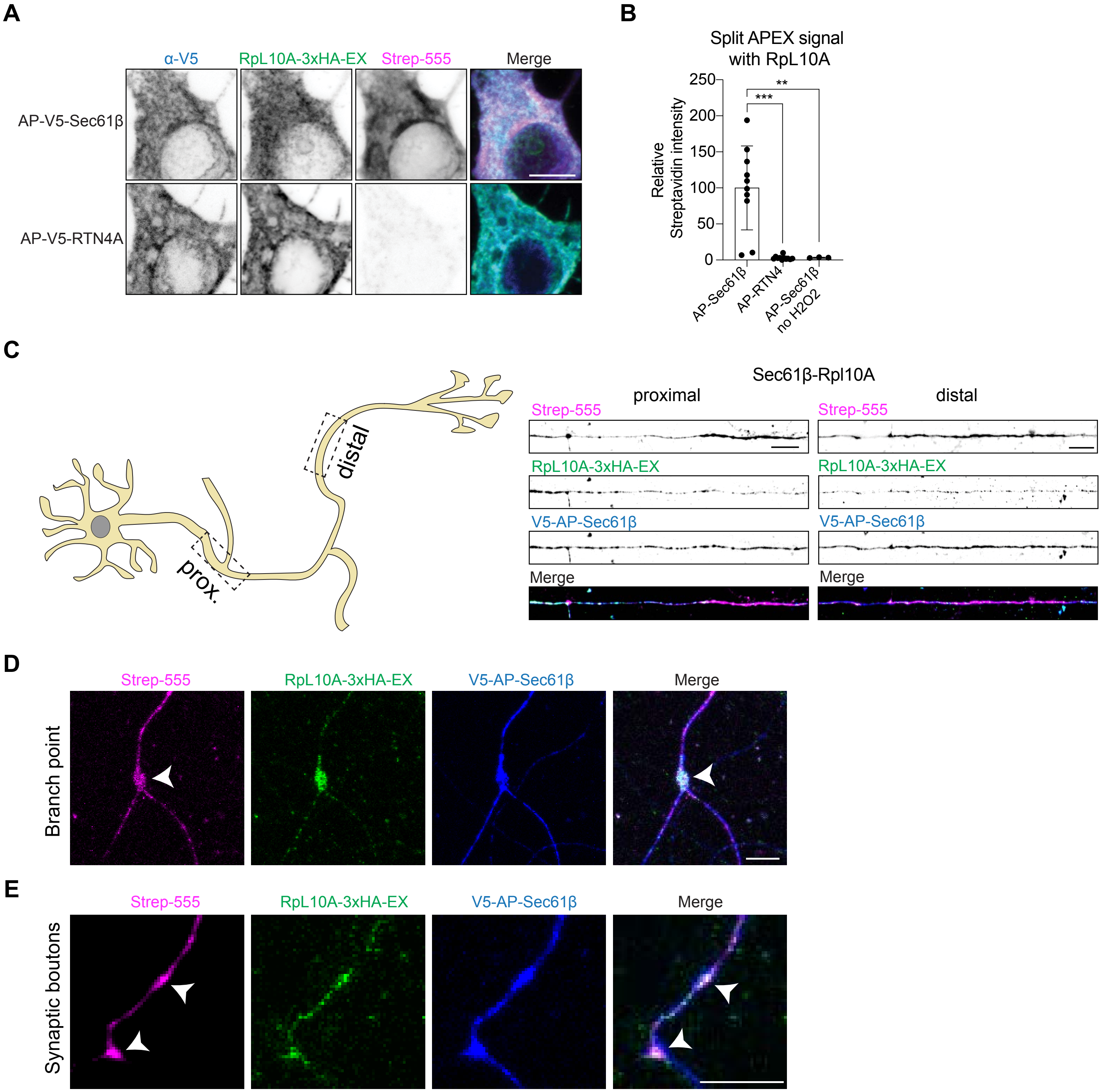

Fig. S3. (Related to Figure 3). Validation of split APEX assay in neuronal soma and split APEX in mature neurons

(**A**) Representative images of split APEX assay in soma from neurons expressing Rpl10A-3xHA-EX and V5-AP-Sec61β (top), or V5-AP-RTN4A as a negative control (bottom). Expression of constructs are visualized with V5 and HA antibodies, and biotinylation is detected with conjugated Strep-555. Scale bars represent 5 µm.
(**B**) Quantification of Strep signal in soma from neurons as in (**A**), and without H2O2 as a negative control for the biotinylation reaction.
(**C**) (left) Schematic representation of a DIV18-21 mature neuron. Boxes indicate regions from which images on the right were taken. (right) Representative images of split APEX in mature neurons (DIV18) showing axonal ER – ribosome interactions in the proximal and distal axon.
(**D-E**) Representative split APEX images of in axon of DIV18 neurons showing enriched signal at a branch point (**D**) and possible synaptic boutons (**E**) as indicated with white arrowheads.
Individual data points each represent a neuron and data are presented as mean values ± SD in (**B**). $^{**}p < 0.01$, $^{***}p<0.001$ comparing conditions to V5-AP-Sec61β using ordinary one-way ANOVA tests. Scale bars represent 10µm in (**A**) and 5µm in (**D-E**).

Figure S4 (related to Figure 4)

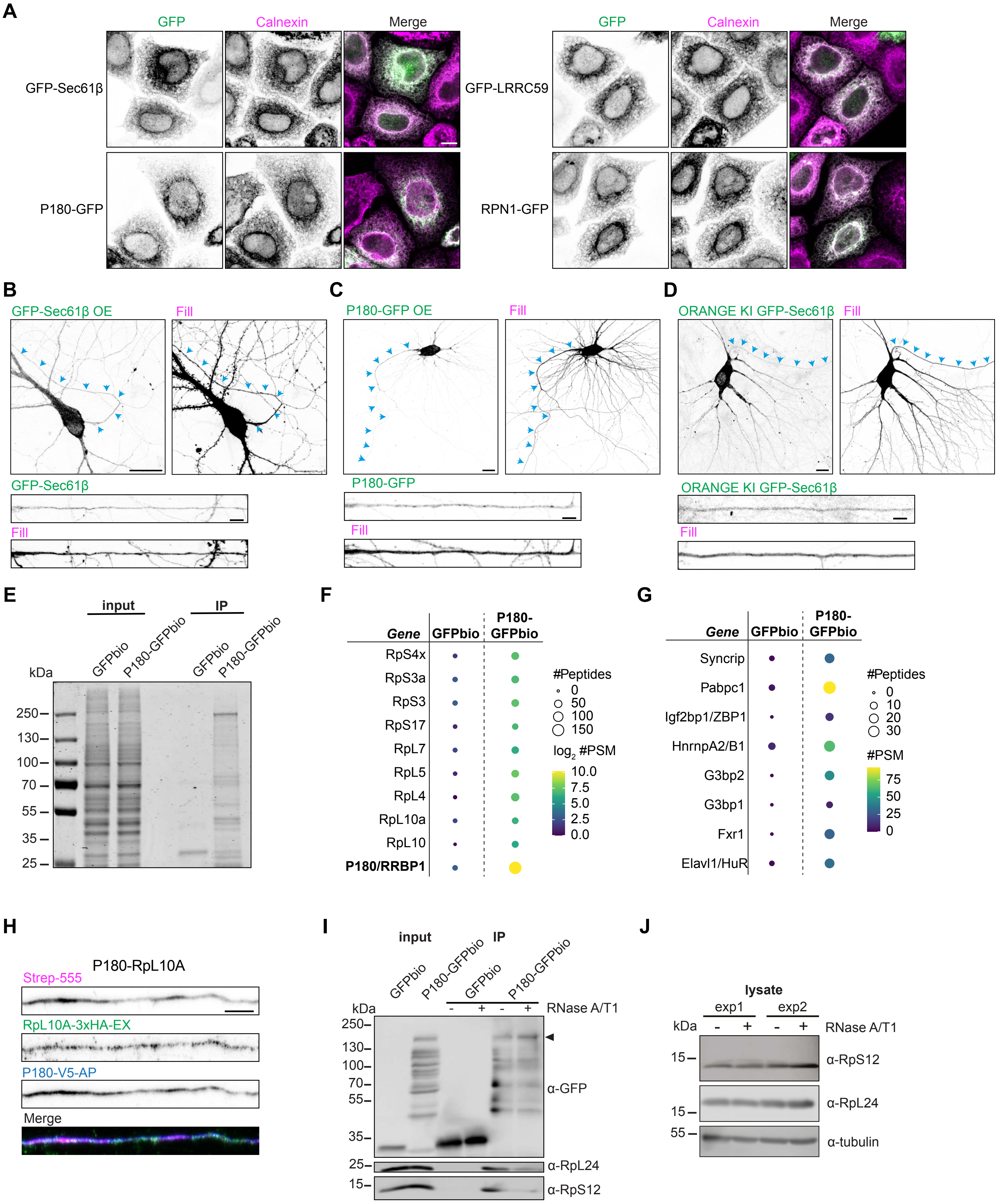

Fig. S4. (Related to Figure 4). Correct ER localization of GFP-tagged constructs, ER protein distribution in mature neurons and ribosomal protein and RNA-binding protein interactions with P180

(**A**) Representative images of GFP-tagged ER protein constructs (as indicated) expressed in HEK293T cells and co-stained with an antibody against the ER marker Calnexin. All constructs show a correct ER localization. Scale bar represents 10 µm.

(**B-D**) Representative images of DIV18-21 neurons transfected with (**B**) GFP-Sec61β (**C**) P180-GFP and (**D**) ORANGE knock-in mNeonGreen-Sec61β together with an mCherry fill. Blue arrowheads indicate the axon of which a straightened zoom is shown below each neuron. Scale bars represents 20µm for soma and 5µm for axons.

(**E**) Silver-stained gel from input and pulldown samples of GFPbio and P180-GFPbio.

(**F**) Scaled representation of ribosomal proteins identified with mass spectrometry after pulldown of GFPbio or P180-GFPbio from HEK293T cells. The size and color of each dot reflect the number of PSMs or peptides identified as indicated in the legend.

(**G**) Scaled representation of RNA-binding proteins identified with mass spectrometry after pulldown of GFPbio or P180-GFPbio from adult rat brain extracts. The size and color of each dot reflect the number of PSMs or peptides identified as indicated in the legend.

(**H**) Representative images of split APEX assay for P180-V5-AP and RpL10A-3xHA-EX in the axon. Expression of constructs are visualized with V5 and HA antibodies, and interactions visualized with conjugated Strep-555. Scale bar represents 5 µm.

(**I**) Western blot analysis of ribosomal proteins after GFPbio or P180-GFPbio pulldown with and without RNAseA/T1 treatment.

(**J**) Western blot images showing RpS12, RpL24 and tubulin protein in lysates with and without RNase A/T1 treatment from two independent experiments. No difference in ribosomal protein level is seen after RNAse A/T1 treatment.

Figure S5 (related to Figure 5)

A

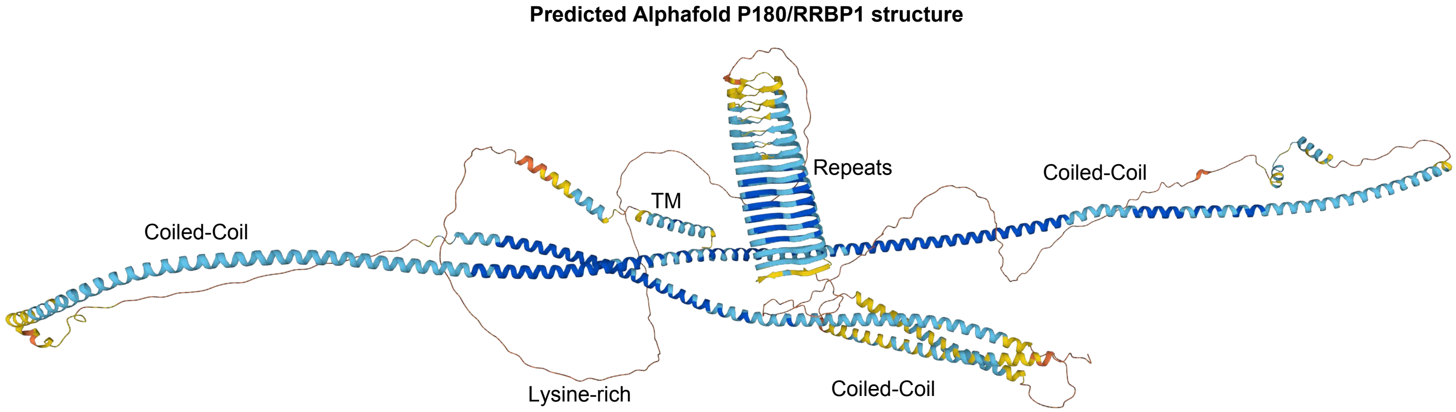

Fig. S5. (Related to Figure 5). Predicted P180/RRBP1 structure and GO analysis of axonally translated P180-enriched mRNAs.

(A) Alphafold2 predicted structure of P180/RRBP1 with each domain annotated.
